# Oxidative Stress-Mediated YAP Dysregulation Contributes to the Pathogenesis of Pemphigus Vulgaris

**DOI:** 10.3389/fimmu.2021.649502

**Published:** 2021-04-19

**Authors:** Yunying Huang, Hana Jedličková, Yang Cai, Ambreen Rehman, Luke Gammon, Usama Sharif Ahmad, Jutamas Uttagomol, Eric Kenneth Parkinson, Farida Fortune, Hong Wan

**Affiliations:** ^1^ Centre for Oral Immunobiology and Regenerative Medicine, Institute of Dentistry, Barts and The London School of Medicine and Dentistry, London, United Kingdom; ^2^ Department of Dermatology, St. Anna University Hospital, Brno, Czechia; ^3^ CB Joint MHNCRL, Hospital and School of Stomatology, Guizhou Medical University, Guiyang, China; ^4^ Phenotypic Screening Facility, Blizard Institute, Barts and The London School of Medicine and Dentistry, London, United Kingdom

**Keywords:** pemphigus vulgaris, yes-associated protein, oxidative stress, reactive oxygen species, cell-cell adhesion, keratinocyte

## Abstract

*Pemphigus Vulgaris* (PV) is a life-threatening autoimmune disease manifested with blisters in the skin and mucosa and caused by autoantibodies against adhesion protein desmoglein-3 (Dsg3) expressed in epithelial membrane linings of these tissues. Despite many studies, the pathogenesis of PV remains incompletely understood. Recently we have shown Dsg3 plays a role in regulating the yes-associated protein (YAP), a co-transcription factor and mechanical sensor, and constraining reactive oxygen species (ROS). This study investigated the effect of PV sera as well as the anti-Dsg3 antibody AK23 on these molecules. We detected elevated YAP steady-state protein levels in PV cells surrounding blisters and perilesional regions and in keratinocytes treated with PV sera and AK23 with concomitant transient ROS overproduction. Cells treated with hydrogen peroxide also exhibited augmented nuclear YAP accompanied by reduction of Dsg3 and α-catenin, a negative regulator of YAP. As expected, transfection of α-catenin-GFP plasmid rendered YAP export from the nucleus evoked by hydrogen peroxide. In addition, suppression of total YAP was observed in hydrogen peroxide treated cells exposed to antioxidants with enhanced cell-cell adhesion being confirmed by decreased fragmentation in the dispase assay compared to hydrogen peroxide treatment alone. On the other hand, the expression of exogenous YAP disrupted intercellular junction assembly. In contrast, YAP depletion resulted in an inverse effect with augmented expression of junction assembly proteins, including Dsg3 and α-catenin capable of abolishing the effect of AK23 on Dsg3 expression. Finally, inhibition of other kinase pathways, including p38MAPK, also demonstrated suppression of YAP induced by hydrogen peroxide. Furthermore, antioxidant treatment of keratinocytes suppressed PV sera-induced total YAP accumulation. In conclusion, this study suggests that oxidative stress coupled with YAP dysregulation attributes to PV blistering, implying antioxidants may be beneficial in the treatment of PV.

## Introduction

Pemphigus Vulgaris (PV) is a severe autoimmune mucocutaneous blistering disease characterized by autoantibodies (PV-IgG) targeting the desmoglein cadherin desmoglein-3 (Dsg3) in the desmosomes of keratinocytes, leading to loss of cell-cell adhesion (acantholysis) (1–5). Originally it was thought to be due to the direct interference of Dsg adhesion through steric hindrance (6–8), later studies uncovered roles for signaling pathways, such as p38 MAPK, SRC, PKC, and ERK upon PV-IgG targeting Dsg3 on keratinocyte surface. Inhibition of these pathways can effectively block blister formation *in vivo* and *in vitro* (2, 9–16). Additionally, several studies have suggested that PV-IgG binds to other surface receptors, resulting in intracellular events, including EGFR, SRC, PKA/C, PLC, mTOR, p38 MAPK, JNK, and activation of cell death machinery and consequently blistering (17–21). Thus, a myriad of intracellular signaling is triggered on PV-IgG binding to keratinocytes. Nonetheless, the molecular mechanism(s) underlying acantholysis remain incompletely understood (5). Further work to depict the molecular alterations underlying the disease process may help identify novel therapeutic strategies to control PV blistering and alleviate the symptoms of the disease.

Yes-associated protein (YAP) is a co-transcription factor involved in cell proliferation, survival and anti-apoptosis (22, 23) and acts as the key downstream effector of the Hippo pathway, an evolutionarily conserved network that plays an integral role in the maintenance of tissue homeostasis (23–25). YAP also functions as a sensor and mediator of many extracellular and intracellular cues, including signaling mediated by reactive oxygen species (ROS) (26). The activity of YAP is negatively regulated by Hippo signaling that facilitates its nuclear export and cytoplasmic accumulation or degradation (23). YAP is also regulated by Hippo-independent mechanisms (25). Dysregulation of YAP can lead to an array of diseases including cancer (25, 27, 28), however, its alteration in PV has not yet been explored. Our recent study has identified that Dsg3 regulates YAP *via* recruiting YAP/phospho-YAP from the nucleus by forming a complex involving plakophilins (29). We also showed that knockdown of Dsg3 caused a reduction of total YAP as well as the *YAP1* target genes with concomitant suppression of cell proliferation, suggesting Dsg3 regulates YAP.

Emerging evidence indicates that oxidative stress contributes to the pathogenesis of pemphigus, with an inverse correlation between the total antioxidant capacity and clinical disease activity (30–33). Nevertheless, it remains elusive whether oxidative stress is a causative factor in PV blistering. In other words, it is unknown whether PV-IgG mediated interruption of Dsg3 has any effect on ROS in keratinocytes. ROS-initiated signaling pathways such as JNK and p38MAPK have been linked to a variety of pathological processes (34–36). Activation of key Hippo components plays a pivotal role in oxidative stress-induced cell signaling (35, 36). Furthermore, YAP has been implicated in regulating antioxidant gene expression *via* complexing with transcription factor FoxO1 and its inactivation by Hippo signaling suppresses FoxO1 activity and decreases antioxidant gene expression (26). In light of our recent findings that 1) Dsg3 exerts a function as an anti-stress protein *via* suppression of p53, ROS and apoptosis and 2) Dsg3 plays a role in regulating YAP (29, 37, 38), we hypothesized that altered YAP caused by oxidative stress might occur in PV. We report here that PV-IgG targeting Dsg3 provoked transient ROS overproduction accompanied by YAP dysregulation, leading to altered cell-cell adhesion and ultimately, blistering. This study provides new insight into the pathogenesis of pemphigus acantholysis and suggests that oxidative stress may be a critical attributing factor in cell damage and impairment of the cell junctional integrity in a YAP-dependent manner in PV.

## Materials and Methods

For the detail description of M+M, please see Supporting Information.

### Cell Lines, PV Sera and Clinical Patient Oral Mucosal Samples

Two immortalized oral and skin-derived keratinocyte cell lines, OKF4 and N/TERT were used in the study. Oral tissue samples of 25 PV patients and 10 normal healthy controls were analyzed. PV sera were obtained from 10 patients; all with informed patient consent and local ethical approval by the Institutional Ethics Committees. The pathogenic activities of PV sera were characterized in keratinocytes in our recent report (37).

### Keratinocyte Culture and Treatment

Non-neoplastic lines N/TERT (derived from the skin) and OKF4 (derived from oral mucosa) used in the study were immortalized normal human keratinocyte cell lines (39, 40) and were maintained in keratinocyte serum-free medium (KSFM) (17005042, Thermo Scientific). For the experiments, in general cells were seeded at approximately 70~80% confluent densities in KSFM for one day before cultured in complete keratinocyte growth medium (KGM) (37). Cells treated with PV sera including dose- and time course experiments, were cultured in calcium-containing (1.2~1.8mM) KSFM for different time periods. Alternatively, cells were treated with the mouse monoclonal antibody AK23 (https://www.mblbio.com/bio/dtl/dtlfiles/D219-3-v8.pdf), alongside the matched isotype control (purified mouse IgG1, κ isotype Ctrl antibody, MG1-45, Biolegend) or hydrogen peroxide (H_2_O_2_), at various concentrations for different time frames according to the experiments before immunofluorescent staining. For antioxidant and inhibitor experiments, cells were pre-treated with drugs for 1 hour before the addition of H_2_O_2_ and incubated for 4 hours. For the dispase assay, 2x10^6^ cells were seeded in 6-well plates and grown in KGM for 2~3 days to reach complete confluence before the assay. Finally, primary human skin keratinocytes of passage 2 were from stock stored in liquid nitrogen in the center. They were thawed and recovered in KSFM before being used for the experiments.

### Immunohistochemistry in PV Specimens

Oral tissue specimens were analyzed using routine immunohistochemistry procedures with rabbit anti-YAP antibody (ab52771, Abcam). The heat-induced epitope retrieval method was used with antigen retrieval buffer EDTA kit (pH=9, Zsbio, China: http://www.zsbio.com/cmsstatic/documents/product/ZLI-9079.pdf), the same method described in our previous report (37). Specifically, slides were transferred to the pressure cooker after boiling. As soon as the cooker reached a maximum temperature for 4 min, the pressure release valve was activated. Once depressurized, the slides were removed and kept on the bench for 10min at room temperature before being cooled on ice and proceeded for YAP immunostaining and image acquisition. Immunochemical positivity was evaluated by two independent pathologists using scoring criteria and defined as negative and positive using a standard method in pathology score.

### Measurement of Reactive Oxygen Species (ROS)

The cellular reactive oxygen species (ROS) levels were determined by incubating cells in 96-well plates with the CellROX Oxidative Stress Reagents (Molecular Probe by Life Technologies), at a final concentration of 5 µM, for 30 minutes at 37°C (38) before image acquisition with an IN Cell Analyzer 2200 (GE Healthcare, UK).

### Plasmid Transfection

Briefly, 2x10^5^ cells were seeded into a 6-well plate overnight before transfected with α-catenin-GFP (gift from Ann Wheeler) or pEGFP-C3-hYAP1 (Addgene Plasmid #17843) or pENTR1A-Yap S127A (Addgene, Plasmid #46050) expression vector along with an empty vector plasmid (pBABE-puro served as the control) using FuGENE HD transfection reagent (E2311, Promega). Cells were re-seeded on coverslips the next day in 24-well plates overnight before being treated with or without hydrogen peroxide according to the experiments.

### Dispase Dissociation Assay

The cell-cell adhesive strength in various conditions was determined by the dispase dissociation assay as described previously (41) (see also **Supporting Information**).

### Luciferase Assay

The YAP luciferase assay was performed using the Bio-Glo™ Luciferase Assay System (G7941, Promega) according to the manufacturer’s instructions. Briefly, GFP-YAP or YAP-S127A transfected cells, alongside empty vector (pBABE-puro) control cells, were seeded in a 24-well plate at ~70% confluence overnight before transfection with 0.25μg YAP luciferase reporter plasmid (Plasmid #34615: 8xTIIC-luciferase, Addgene) per well using FuGENE HD transfection reagent. After 24 hours, cells were washed with PBS before the luciferase assay.

### YAP siRNA Transfection

Transient YAP siRNA transfection was performed in N/TERT cells. Two siRNA sequences were purchased from Dharmacon (Colorado, USA) ON-TARGETplus siRNA-7 J-012200-07 and siRNA-8 J-012200-08, Human YAP1 NM-001130145 (siRNA-7: GGTCAGAGATACTTCTTAA [referred as siRNA-1] and siRNA-8: CCACCAAGCTAGATAAAGA [referred as siRNA-2]), along with the scrambled control provided by the same company. In brief, 2x10^6^ cells were seeded into a 100 mm dish overnight and then were transfected with either scrambled or specific siRNA at a final concentration of 50 nM in KSFM using DharmaFECT 1(T-2001-02, Dharmacon) following the manufacturer’s instructions. The next day, cells were harvested with 0.25% Trypsin/EDTA and re-plated at approximately 70~80% confluent densities for Western blotting or immunofluorescence analysis as described previously (29, 37).

### Statistical Analysis

For multiple comparisons and the comparison of two groups, the one-way analysis of variance (ANOVA) and the two-tailed Student *t-test* were used, respectively to obtain p values. Values less than 0.05 were considered statistically significant, *i.e.* *p<0.05, **p<0.01, ***p<0.001, and ****p<0.0001. For comparison of PV patient samples and healthy controls, the Chi-Square Test was used to obtain the p-value.

## Results

### YAP Steady-State Protein Levels Increase in PV

**Figure 1:**
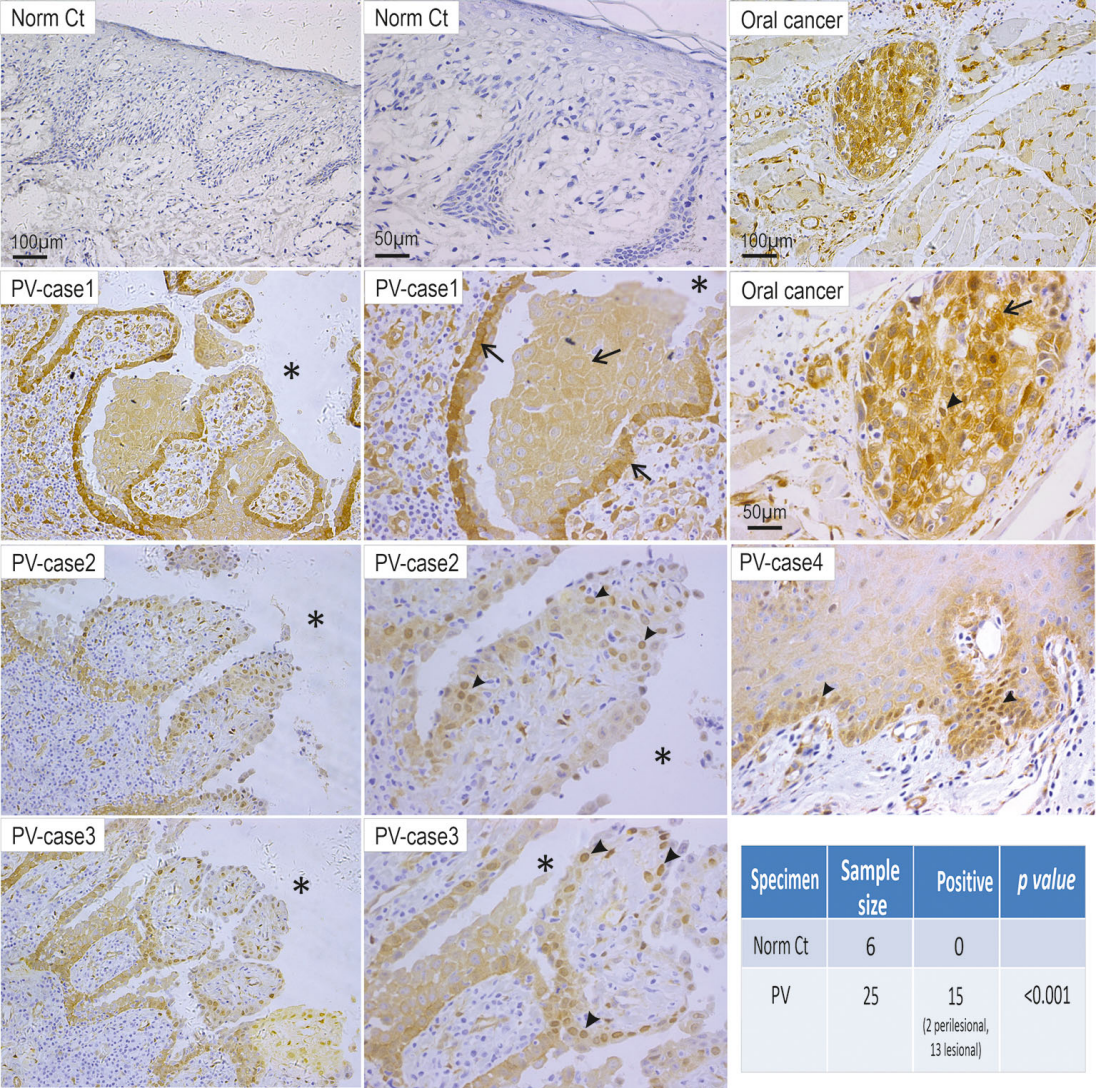
Elevated YAP steady state protein levels are detected in the oral mucosa of patients with PV. Twenty-five PV cases and six healthy individual controls were analyzed by immunohistochemistry for YAP. Positivity of YAP staining with increased nuclear (arrowheads) and cytoplasmic (arrows) signals was detected in cells surrounding the blisters (13 cases) and perilesional regions (2 cases) in 15 PV patients, whereas the healthy control samples (Norm Ct) showed little or no staining. Asterisks indicate the locations of blisters. Oral cancer was used as a positive control here that showed increased YAP in both the nuclei and cytoplasm. Chi-Square statistic was used to obtain the p-value for comparison between PV and healthy control groups, shown in the table. Scale bars are displayed in the images.

Recently, we have reported that Dsg3 is capable of regulating two fundamental pathways, p53 and YAP that control cell proliferation, differentiation and cell fate decision (29, 37). However, it remains unknown whether the alteration of YAP occurs in PV. Given our previous finding that Dsg3 depletion caused a reduction of YAP and phospho-YAP, it was speculated that antibodies targeting Dsg3 in PV may cause YAP attenuation. To address this question, first, we performed immunohistochemistry for YAP in oral mucosal tissue specimens of 25 PV patients alongside 6 healthy controls. Unexpectedly, we observed an increase of YAP in 15 of 25 PV cases compared to control samples, which displayed almost negative staining (p<0.001, **Figure 1**). Positivity in the YAP staining was prevalent in the cytoplasm of cells surrounding the blisters in some cases (5 of 15 PV cases) or the nuclei of cells in others (10 of 15 PV cases). Notably, positive nuclear YAP was detectable in perilesional regions in 2 out of 15 YAP positive PV samples (Case4 **Figure 1**). These observations prompted us to hypothesize that aberrant YAP steady-state protein levels might attribute, at least in part, to the onset of pemphigus acantholysis.

### Augmented YAP Accumulation in Keratinocytes Treated With PV Sera and the Pathogenic Monoclonal Antibody Targeting Dsg3

**Figure 2:**
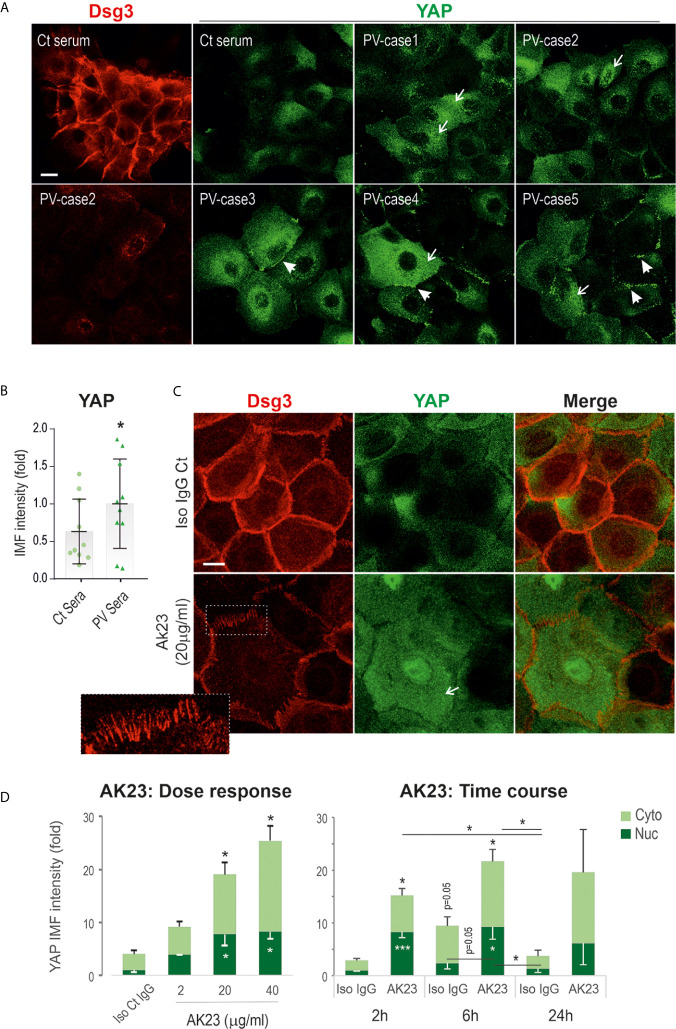
Treatment of keratinocytes with PV sera or the pathogenic monoclonal antibody against Dsg3 results in increased YAP expression. **(A)** Confocal images of N/TERT cells double-labeled for Dsg3 (5H10, red) and YAP (green). Cells were seeded on coverslips at approximately 80% confluent densities (~2.5x10^5^/well) one day before being treated with PV sera (40%) in KSFM (Ca^++^ 90µM) alongside control serum overnight. Then, the medium was replaced with KSFM with normal calcium (1.8mM) plus PV sera at the same concentration of 40% and cultured for further 5 hours (24 hours PV sera in total) before fixation. Coverslips were immunostained for the indicated proteins. Increased cytoplasmic YAP (arrows) with aggregations at the cell borders (arrowheads) were shown in PV serum treated cells coupled with Dsg3 reduction. **(B)** Image quantitation of YAP staining shown in **(A)** (n=10 PV patients’ sera, 2 experiments, the comparison was made by two-tailed Student *t-test*, *p<0.05). **(C)** N/TERT cells of ~80% confluence grown for one day were treated with AK23, the pathogenic monospecific antibody against Dsg3, in KGM for 6 hours and dual labeled for Dsg3 (5H10) and YAP. The insert showed the characteristic linear arrays of Dsg3 staining (red) at the cell border, the feature of anti-Dsg3 mediated disruption as described previously (42). Elevated YAP levels were shown in both the nuclei (Nuc) and cytoplasm (Cyto) of AK23 treated cells compared to the counterpart treated with isotype IgG1 control (Iso Ct IgG). Reduction of Dsg3 staining was observed in cells treated with AK23. **(D)** Image quantification for the dose and time-course experiments (normalized to control nuclear signal that was arbitrarily set as 1), with AK23 (2 µg/ml used in the time course study) (n=4 fields/sample, 2 experiments for dose and 3 experiments for time course, mean ± SEM, one-way ANOVA was used to obtain the p values (*p<0.05, ***p<0.001). Data were representative of at least three independent experiments. Scale bars, 10µm.

To validate the immunohistochemical findings, we studied two immortalized keratinocyte cell lines N/TERT (skin-derived) and OKF4 (oral-derived) treated with PV sera (including 10 patients) with their pathogenicity towards Dsg3 depletion characterized previously (37), alongside with AK23, a mouse monoclonal antibody (mAb) against Dsg3 (7). Cells at confluent densities on coverslips were treated with PV sera (40% PV sera) for 24 hours before immunostaining for YAP and Dsg3 (**Figure 2A**). In contrast to control serum-treated cells that displayed strong Dsg3 membrane distribution and faint diffuse YAP cytoplasmic staining (the characteristics of YAP in confluent keratinocytes (29), cells treated with PV serum showed augmented YAP signals, especially in the cytoplasm (arrows**** **Figure 2A**). Intriguingly, many linear dense fragments of YAP were observed at the cell borders (****arrowheads **Figure 2A**), especially in the populations that showed significant loss of Dsg3 staining. Overall, as reported previously (37), a reduction of Dsg3 was detected in PV serum-treated samples. Image quantitation of YAP immunofluorescence agreed with this observation and indicated a significant increase in PV serum-treated cells compared to the control group (**Figure 2B**). YAP nuclear staining was detectable in the populations of N/TERT seeded at the colony densities exposed to PV sera, however, this feature was hardly seen in control cells (**Figure S1A**). Similar results with elevated YAP were also observed in the OKF4 cell line (**Figure S1B**) that appeared more sensitive to PV sera than N/TERTs since 50% PV serum-treated wells had the cells lost after fixation, potentially due to cytotoxicity or the activation of the intracellular apoptosis machinery that leads to cell detachment from the substrate.

Subsequently, we treated cells with the pathogenic mAb AK23 using increasing doses over various time frames. Again, cells treated with AK23 also showed increased YAP staining in both the nuclei and cytoplasm in N/TERTs in a time- and dose-dependent manner, especially within 6 hours (**Figures 2C, D**). Close inspection observed a lack of YAP linear fragments at the cell border in AK23 treated cells that differed from PV sera treated cells as described above. As expected, reduction of Dsg3 at the cell junctions with typical organized linear arrays as described previously (42) was observed in AK23 treated cells (insert **Figure 2C**). A similar finding with enhanced nuclear YAP was also seen in OKF4 cells treated with AK23 for 24 hours compared to the counterpart treated with isotype IgG1 (**Figure S1C**). Immunostaining for α-catenin, a negative regulator of YAP (43, 44), revealed a significant reduction in AK23 treated cells, especially at the early time points of 2 and 6 hours (**Figure S1D**).

**Figure 3:**
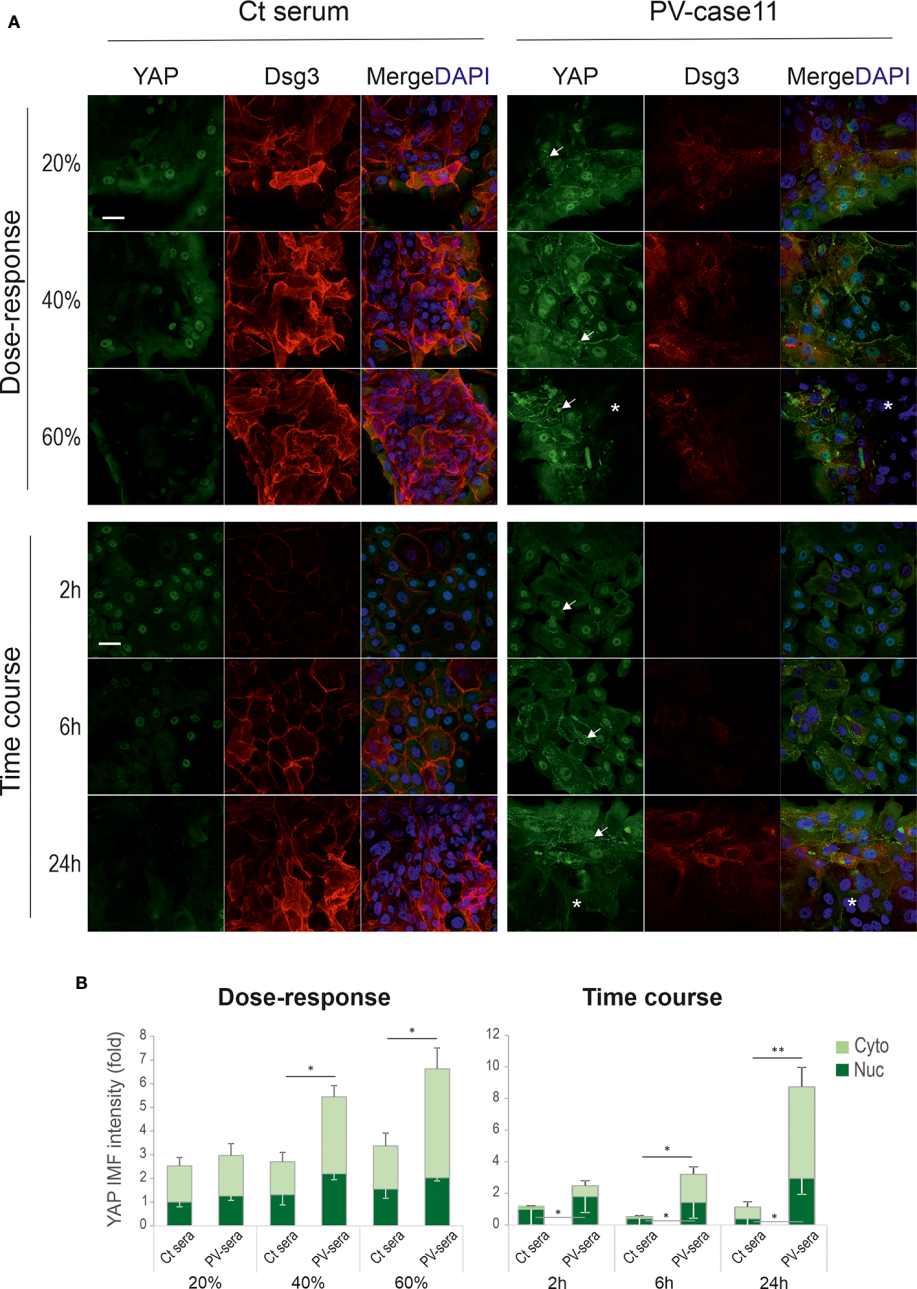
Primary human skin keratinocytes treated with PV serum also show aberrant YAP expression. **(A)** Confocal images of primary human skin keratinocytes double labeled for YAP and Dsg3. Cells were seeded at ~60% confluent densities (~2.5x105/well) in KSFM for one day before treated with different doses of PV sera at 20%, 40% and 60% for 24 hours (top panels) or 40% for 2, 6 and 24 hours (bottom panels), respectively, in calcium-containing KSFM. For the dose experiment, cells were exposed to PV serum in KSFM (low Ca++) overnight. Then the serum media were changed to high calcium-containing (1.8mM) KSFM, and cells were incubated for additional 6 hours before fixation (total 24 hours). For the time-course experiment, cells were treated with PV serum in calcium-containing KSFM (Ca^++^, 1.8mM) for various time points before fixation. Notably, cells were sensitive to calcium-containing medium and with a greater degree than N/TERT cells. In control cells, strong YAP nuclear staining was displayed at the edge of the colonies coupled with faint diffuse cytoplasmic signals. In contrast, cells treated with PV serum showed increased YAP with membrane distribution and cytoplasmic aggregates (arrows). Reduced YAP signals were detected in the colonies’ central areas, especially in samples of 60% PV sera treated cells and 40% PV sera treated cells for 24 hours (asterisks). Reduction of Dsg3 staining was evident in PV serum treated cells compared to the respective controls. **(B)** Image quantification for those shown in A (normalized to control nuclear signal that was arbitrarily set as 1) (n=5 fields/sample, mean ± SEM, *p<0.05, **p<0.01). Scale bar, 20µm.

To consolidate the above findings, we analyzed YAP and Dsg3 expression in primary human skin keratinocytes of early passage on coverslips and cells were seeded at approximately 60% confluence before being treated with PV sera at different dosages (*i.e.* 20%, 40% and 60%) for various time frames (*i.e.* 2, 6 and 24 hours) prior to fixation. Again, similar findings with YAP membrane distribution and cytoplasmic aggregates coupled with Dsg3 reduction were observed in PV serum treated cells in contrast to the respective controls (arrows **Figure 3A**). Elevated YAP expression in PV sera treated cells appeared to be in a time and dose-dependent manner (**Figure 3B**), especially at the edge of the colonies, whereas control cells showed an opposite trend with faint YAP staining in 60% PV sera treated cells or at 24 hours’ time point. Collectively, these *in vitro* data seemed to substantiate *in vivo* findings (**Figure 1**) and suggest that PV-IgG targeted disruption and depletion of Dsg3 promotes YAP accumulation accompanied by the suppression of α-catenin in keratinocytes or *vice versa*. The enhanced YAP nuclear signals were likely reflecting the degree of disruption in cell junctions mediated by PV-IgG. Apparently, this result was in contrast to our initial prediction of YAP downregulation caused by PV antibodies that targeting Dsg3.

### Transient ROS Overproduction Is Detected in Keratinocytes Treated With PV Sera and AK23

**Figure 4:**
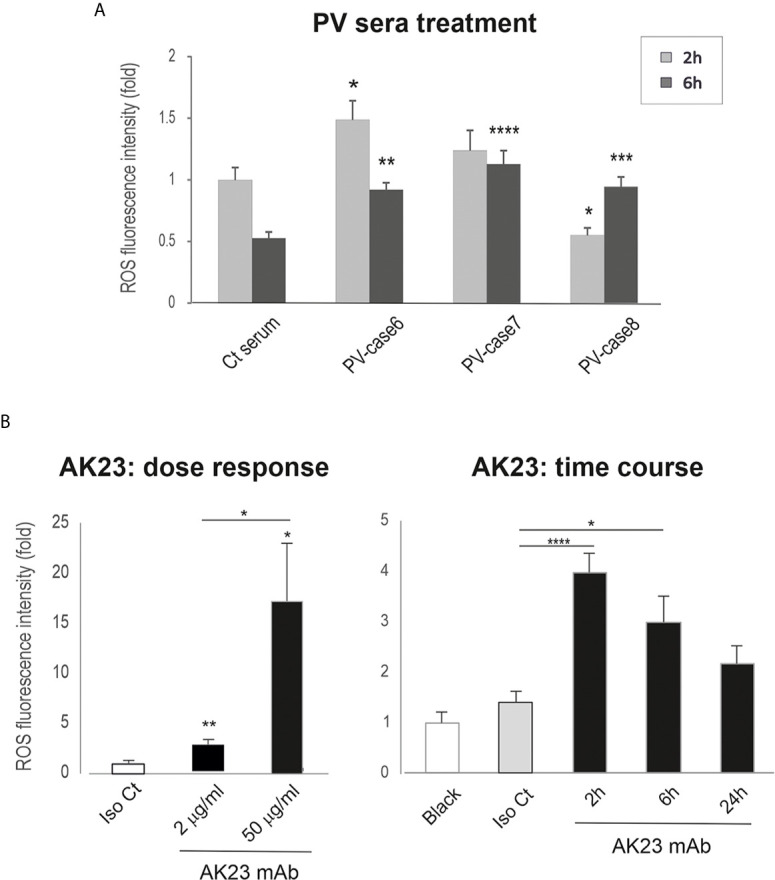
Keratinocytes treated with PV sera and AK23 against Dsg3 shows transient ROS overproduction. Oral keratinocytes OKF4 seeded at approximately 70% confluent density in 96-well plate (*i.e.* ~1x10^5^ cells per well) in KSFM overnight before treated with PV sera (40% in KGM) **(A)** (pooled from 2 out of 3 independent experiments) and AK23 **(B)** (at least 2 experiments) with elevated concentrations or for increasing periods up to 24 hours. Iso Ct: Isotype control IgG1. Cells were then incubated with CellRox reagent (5µM) for 30 minutes before brief washing with PBS followed by image acquisition with an INCA 2200 Analyzer system straightaway. Transient increase of ROS was detected in cells treated with PV sera or AK23 compared to the respective controls, with a dose-dependent response for AK23 (n=25 automated fields/well, mean ± SEM, one-way ANOVA, *p < 0.05, **p < 0.01, ***p < 0.001, ****p < 0.0001). Of note, a few wells treated with PV sera had a large proportion of cells lost and were excluded from the analysis. The data shown were representative of three independent experiments.

Our recent reports show that Dsg3 exerts a function in the suppression of p53 and ROS (37, 38). These findings led us to hypothesize that PV-IgG targeting Dsg3 may trigger oxidative stress in keratinocytes with elevated ROS production that, in turn, has an impact on the integrity of intercellular junctions leading to acantholysis. In line with this notion, a few studies have shown that increased oxidative stress is associated with PV clinical activity (30–32). In addition, studies suggest that elevated ROS disrupt cell adhesion and the epithelial barrier function *via* destabilizing intercellular junctions, the effect that can be inhibited by antioxidants (45–48). To determine whether PV-IgG is capable of inducing ROS, we analyzed the ROS levels in cells treated with PV sera and AK23, respectively, using CellRox Oxidative Stress Reagents (38). In this case, OKF4 cells seeded in 96-well plates overnight were subjected to the treatment with PV sera or AK23 at elevated concentrations or for increasing periods up to 24 hours. The cells were then incubated with CellRox for 30 minutes before image acquisition with the INCell Analyzer system, followed by image quantitation. The resulting data indicated a transient increase of ROS in cells treated with PV sera or AK23 in a time- and dose-dependent manner, compared to the respective controls (**Figures 4A, B**).

### Hydrogen Peroxide Alters Junctional Protein Expression and Promotes YAP Cytoplasmic Accumulation

**Figure 5:**
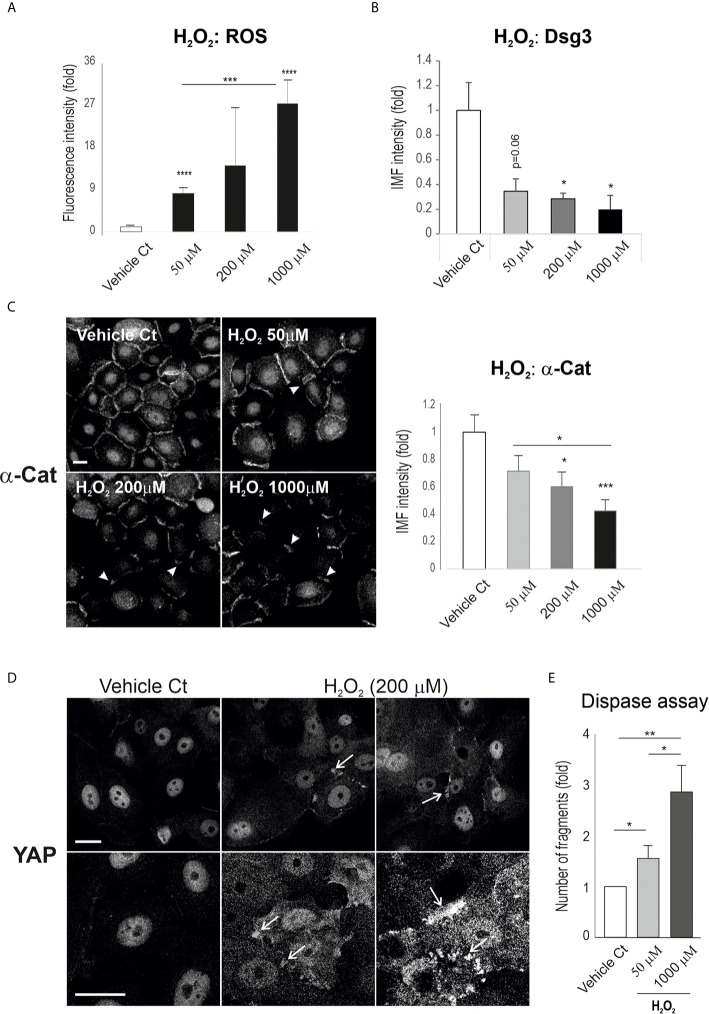
H_2_O_2_ treatment of keratinocytes causes a reduction of cell junction proteins with concomitant YAP accumulation in the cytoplasm and cell periphery. **(A)** A dose-dependent increase of ROS was detected in the cell populations treated with H_2_O_2_. Oral keratinocytes OKF4 were seeded at 5x10^4^ per well in KSFM overnight to reach ~70% confluent densities in 96-well plate and then exposed to H_2_O_2_ in KGM at elevated concentrations for 2 hours before treatment with CellRox reagent (5µM) for 30 minutes for ROS measurement. Images were acquired immediately with an INCA 2200 (n=14~16, at least 2 experiments) and analyzed with Developer Toolbox software. **(B**, **C)** Analysis of cell adhesion proteins Dsg3 and α-catenin indicated a dose-dependent decrease in their expression in cells treated with H_2_O_2_. Dsg3 staining was performed on coverslips with similar cell densities (5H10 mAb, n=5 fields/sample, 2 experiments) while α-catenin staining was conducted in 96-wells (n=25 automated images, 2 experiments, mean ± SEM). **(D)** Confocal images of YAP staining in cells of similar densities, treated with H_2_O_2_ at one dosage for 2 hours. Enhanced YAP expression was evident, especially at the cell borders with marked accumulation (arrows, representative of 2 experiments). **(E)** Dispase dissociation assay in cells treated with H_2_O_2_ at the indicated concentrations along with PBS vehicle control. Cells were seeded at confluent densities in 6-well plates and grew in KGM for 2~3 days till the junctions were well-established. Then, cells were subjected to H_2_O_2_ treatment for 4 hours. The epithelial sheets were released by dispase (2.4 units/ml) after approximately 20 minutes. Fragmentation of epithelial sheets was induced by mechanical stress followed by image acquisition. The number of fragments in each sample was determined with ImageJ (duplicate per experiment, 3 experiments, Mean ± SEM, one-way ANOVA was used for comparison between groups. *p<0.05, **p<0.01, ***p<0.001, ****p<0.0001). Scale bars, 10µm. Nuc, nucleus; Cyto, cytoplasm.

To establish the relationship between ROS and YAP, we examined the effect of ROS on YAP and the adhesion proteins α-catenin and Dsg3. OKF4 cells were seeded in a 96-well plate at approximately 70~80% confluence and were subjected to treatment with H_2_O_2_ at various concentrations, *i.e.* 50, 200, 1000 µM, for 2 hours. This was followed by CellRox Reagent treatment for 30 minutes before image acquisition and analysis. First, the induction of ROS by H_2_O_2_ was confirmed that demonstrated a dose-dependent augmentation of ROS in H_2_O_2_ treated samples (**Figure 5A**). In parallel, immunofluorescence for Dsg3 and α-catenin was performed in cells treated with H_2_O_2_ which showed convincingly a dose-dependent reduction in both proteins compared to vehicle controls (**Figures 5B, C**), demonstrating the effect of ROS overproduction on the disruption of the junction assembly proteins that resemble the Dsg3 loss induced by PV-IgG. Close inspection revealed the linear junctional staining pattern of α-catenin in control cells became broken and/or fragmented in H_2_O_2_ treated cells, with the degree of disruption coinciding with the concentrations of H_2_O_2_ (arrowheads **Figure 5C**).

Next, the expression and subcellular distribution of YAP was analyzed in cells seeded on coverslips. Confocal microscopy in H_2_O_2_ treated cells depicted pronounced YAP accumulation, especially in the cytoplasm, with many aggregates detected adjacent to the plasma membrane and these findings mimicked the observations in PV-IgG treated cells as well as in some clinical samples of PV patients (**Figure 1**, **Figure 2A**). The control cells, however, showed predominantly YAP nuclear staining.

To confirm the effect of these molecular changes on cell-cell adhesion integrity and strength, we performed the dispase assay in both OKF4 and N/TERT lines. In this functional study, confluent cells grown in 6-well plates were treated with H_2_O_2_ at the various concentrations for 2 hours before being treated with dispase (2.4 unit/ml), followed by pipetting to induce fragmentation. Finally, the number of fragments in each sample were scored and presented (**Figure 5E** for OKF4, **Figure S2** for N/TERT). Consistently, both cell lines subjected to H_2_O_2_ exposure showed a dose-dependent increased fragmentation, suggesting that elevated ROS cause changes in molecules such as YAP, Dsg3 and α-catenin that lead to disruption of the cell junctions and intercellular adhesions.

### Expression of Exogenous α-Catenin or Antioxidant Can Mitigate Hydrogen Peroxide-Induced YAP Accumulation

**Figure 6:**
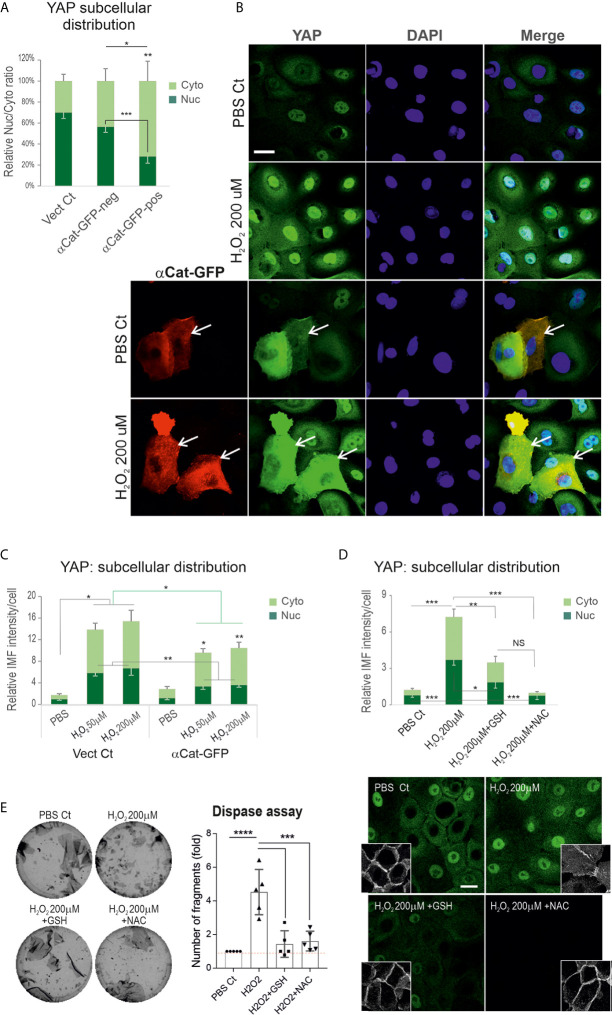
Expression of exogenous α-catenin or treatment of keratinocytes with antioxidants can rescue H_2_O_2_ inducted YAP nuclear accumulation with concomitant enhancement of the cell junctional integrity. **(A)** Analysis of YAP expression and distribution in GFP+ (indicating α-catenin-GFP transfection) and GFP- cells (α-catenin-GFP un-transfected cells), respectively. Cells transfected with empty vector served as the control. Expression of α-catenin-GFP caused a reduction of nuclear YAP. **(B)** Confocal images of α-catenin-GFP plasmid transfected or mock control N/TERTs seeded at 2.5x10^5^ per well to reach ~70% confluent densities. Next day cells were treated with or without H_2_O_2_ (only one dosage was displayed) for 2 hours and doubled labeled for YAP (green) and GFP (red). YAP nuclear exclusion was evident in cells with α-catenin-GFP expression. **(C)** Image quantitation in different conditions shown in B (n=5 fields/coverslip, representative of 2 independent experiments, Mean ± SEM, normalized to PBS control nuclear signal that was arbitrarily set as 1). The green line indicates comparison of total fluorescent intensity between control and α-catenin-GFP transfected cells, whereas the black lines were comparisons for cytoplasmic (Cyto) and nuclear (Nuc) signals, respectively. **(D)** YAP expression in cells of ~70% confluence, treated with one dosage of H_2_O_2_ in the presence and absence of GSH (10μM) and NAC (2mM) for 2 hours (n=13, pooled data from 3 independent attempts, Mean ± SEM). Cells were treated with antioxidants 1 hour before H_2_O_2_ addition. Confocal images for YAP and Dsg3 (inserts, stained with 5H10 mAb) were displayed underneath. **(E)** Dispase assay in different conditions as shown in D. Cells of 100% confluence grown in KGM were treated with H_2_O_2_ in the presence and absence of GSH (10μM) and NAC (2 mM) for 4 hours (n=5, pooled from 3 independent experiments, with one in triplicate, Mean ± SD),. One-way ANOVA was used to obtain all p values. *p<0.05, **p<0.01, ***p<0.001, ****p<0.0001, and NS indicates no significance. Scale bars, 10µm.

Convincing evidence shows α-catenin as a key negative regulator of YAP and its depletion results in YAP nuclear relocation with accelerated keratinocyte proliferation *in vitro* and *in vivo* (43, 44, 49). We hypothesized that PV-IgG triggered augmentation of YAP might be caused by decreased α-catenin mediated by ROS overproduction. Consistent with this possibility, we had already demonstrated changes in the expression levels and junction assembly of α-catenin in cells treated with H_2_O_2_ coupled with YAP cytoplasmic accumulation (**Figures 5C, D**), indicating an adverse effect of ROS on α-catenin and the positive impact on YAP dysregulation. To consolidate this finding, we performed a rescue experiment in N/TERTs with transfection of the α-catenin-GFP vector before H_2_O_2_ exposure at various concentrations. Cells without transfection served as the control here. First, we analyzed YAP expression levels and subcellular distribution in GFP-positive and negative cells and confirmed that GFP-positive cells (indicating expression of exogenous α-catenin) had significantly low nuclear and high cytoplasmic YAP compared to GFP-negative cells (**Figure 6A**) that ascertained the reciprocal crosstalk of these two molecules. Then, cells transfected with or without α-catenin-GFP were treated with H_2_O_2_ for 2 hours before double staining with anti-YAP and anti-GFP antibodies. The resulting data indicated that expression of α-catenin was able to inhibit ROS induced YAP nuclear accumulation as well as the total YAP levels compared to the respective controls (**Figures 6B, C**), indicating α-catenin can alleviate ROS mediated YAP nuclear accumulation.

Next, we tested whether antioxidants have a similar effect. Cells seeded on coverslips were treated with glutathione (GSH, 10μM) and N-acetyl cysteine (NAC, 2mM), respectively, for 1 hour before addition of H_2_O_2_ and incubated for further 2 hours. YAP immunofluorescence demonstrated both NAC and GSH significantly block the increased YAP induced by H_2_O_2_ as well as the disrupted Dsg3 at the junctions (**Figure 6D**). Again, a similar effect was observed in OKF4 (**Figure S3A**). Finally, the cell-cell adhesion was analyzed by the dispase assay in both cell lines treated with H_2_O_2_ in the presence and absence of antioxidants GSH and NAC alongside the vehicle control. Consistently, the H_2_O_2-_induced weakening of cell junctions was remarkably inhibited by antioxidants, with a significant reduction of fragments with the number comparable to control as opposed to H_2_O_2_ treated alone (**Figure 6E**, **Figure S3B**). Of note, the reduced fragments correlated with enhanced cell-cell adhesion and integrity.

### Expression of Exogenous YAP Perturbs Junction Assembly of the Adhesion Proteins, While YAP Silencing Causes Enhanced Expression of Dsg3 and α-Catenin

**Figure 7:**
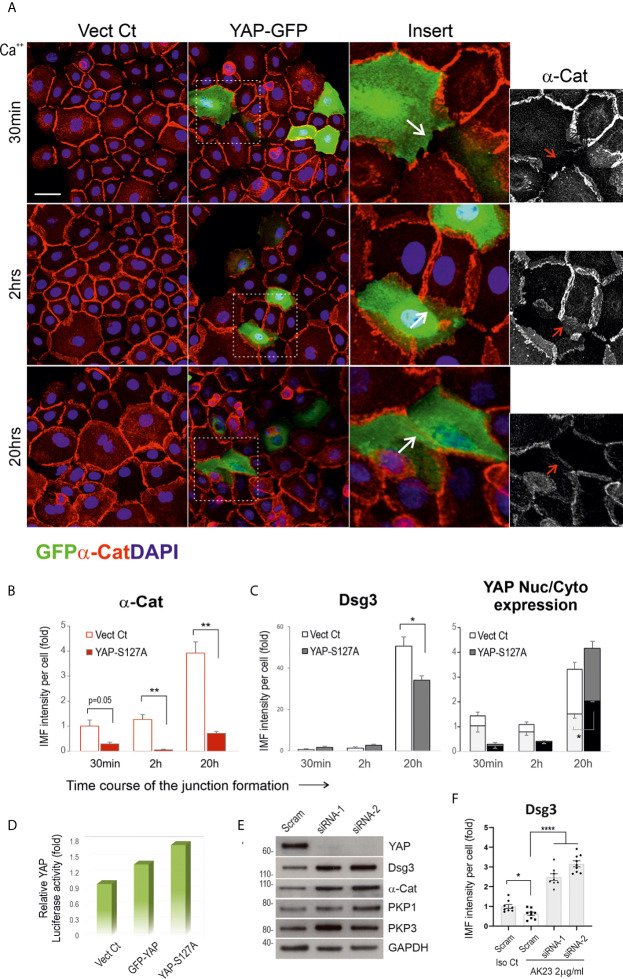
Expression of exogenous YAP causes disruption in the junctional assembly of α-catenin and Dsg3. **(A)** Confocal images of N/TERTs transfected with GFP-YAP or empty vector plasmid (Vect Ct), double-stained for GFP (green) and α-catenin (red). Cells were seeded at 2.5x10^5^ per well on coverslips in a 24-well plate in KSFM to reach ~70% confluence. The next day, the mediums were changed to KGM and cells were incubated for various periods before fixation (2 experiments). Inserts showed enlarged fields of the square boxes with GFP positive cells in which disruption of α-catenin at cell junctions was evident. **(B)** Image quantitation for α-catenin staining in cells transfected with YAP-S127A plasmid or Vect Ct and cells were treated in the same way as described in **(A)**. A significant reduction in α-catenin expression was detected at 2 hours and 20 hours, especially at the later time point, compared to controls (n=5 fields/sample, 2 experiments, **p < 0.01). **(C)** Image quantitation for Dsg3 (with 5H10) and YAP staining in a parallel set of coverslips that indicated increased nuclear YAP in cells transfected with YAP-S127A compared to Vect Ct cells (n=5 fields/sample, 2 experiments, *p < 0.05). A reduction of Dsg3 was also detected at 20 hours’ time point in YAP-S127A transfected cells compared to control (*p < 0.05). **(D)** YAP luciferase assay indicated a trend of increased luciferase activities in both GFP-YAP and YAP-S127A transfected cells compared to Vect Ct. **(E)** Western blotting for the indicated proteins in N/TERTs transfected with either scrambled (Scram) or siRNA-1/2. Increased expression of cell adhesion proteins was shown in YAP knockdown cells compared to controls (at least 3 experiments, unpublished data in other cell lines). **(F)** Image quantitation of Dsg3 staining in N/TERTs transfected with scrambled (Scram) or siRNA-1/2 and treated with isotype IgG control (Iso Ct) and AK23 (2µg/ml), respectively. 1x10^5^ siRNA transfected cells were seeded in 96-well in KSFM overnight before IgG treatment in calcium-containing (1.2mM) KSFM for 24 hours. It showed YAP depletion resulted in a 2~3-fold increase of Dsg3 expression compared to Scram control cells treated with Iso Ct IgG, which blunted the effect of AK23 induced Dsg3 reduction (n=8~9 fields/sample, *p < 0.05, ****p < 0.0001). Scale bars, 20µm.

To determine whether YAP overexpression can perturb junction formation, we transfected N/TERT cells with two different YAP plasmids, i.e. pEGFP-C3-hYAP1 (GFP-YAP) and pENTR1A-Yap S127A (YAP-S127A) alongside empty vector control (Vect Ct) overnight. The transfected cells were harvested and plated on coverslips in 24-well plate at ~70% confluence in KSFM. The next day, the medium was replaced with KGM (contained normal Ca++ at 1.8 mM). Cells were incubated for various periods up to 20 hours before fixation and double stained for α-catenin/GFP in one set and Dsg3/YAP in another (**Figures 7A–C**). YAP activities in three different conditions were determined by YAP luciferase assay (**Figure 7D**). Confocal microscopy was performed in GFP-YAP transfected cells and Vect Ct sample, and close inspection indicated disruption of junctions in GFP positive cells in contrast to adjacent internal control cells with negative GFP signals as well as Vect Ct cells (****arrows in inserts **Figure 7A**). Quantitation of α-catenin expression showed a significant reduction at both 2 and 20 hours’ time points compared to the respective controls (**Figure 7B**). A similar finding was also observed in Dsg3 staining in YAP-S127A transfected cells with increased YAP nuclear signals (**Figure 6C**). To address whether YAP depletion results in an inverse effect, we performed YAP knockdown in N/TERTs using two specific siRNA hits. Significant YAP silencing was achieved with both hits compared to scrambled siRNA treated cells, and increased expression of cell adhesion proteins such as Dsg3, α-catenin and PKP1/3 was detected in YAP knockdown cells (**Figure 7E**). Treating cells with AK23 alongside isotype IgG demonstrated that YAP silencing was able to eliminate the pathogenicity of AK23 mediated Dsg3 depletion (**Figure 7F**). In this case, the siRNA transfected cells were seeded at ~70% confluence in a 96-well plate and treated with AK23 at a concentration of 2µg/ml in calcium-containing (1.2mM) medium for 24 hours before Dsg3 staining, image acquisition and processing. These results collectively suggest that increased YAP expression elicits perturbation or retardation in cell junction assembly, with its depletion renders the opposite effect with protection in cell cohesion against the anti-Dsg3 antibody, with even 2~3-fold increase of Dsg3 expression levels compared to scrambled siRNA transfected cells treated with isotype IgG.

### Inhibition of p38MAPK, JNK and PKC Alleviates YAP and Its Nuclear Accumulation Induced by Hydrogen Peroxide

**Figure 8:**
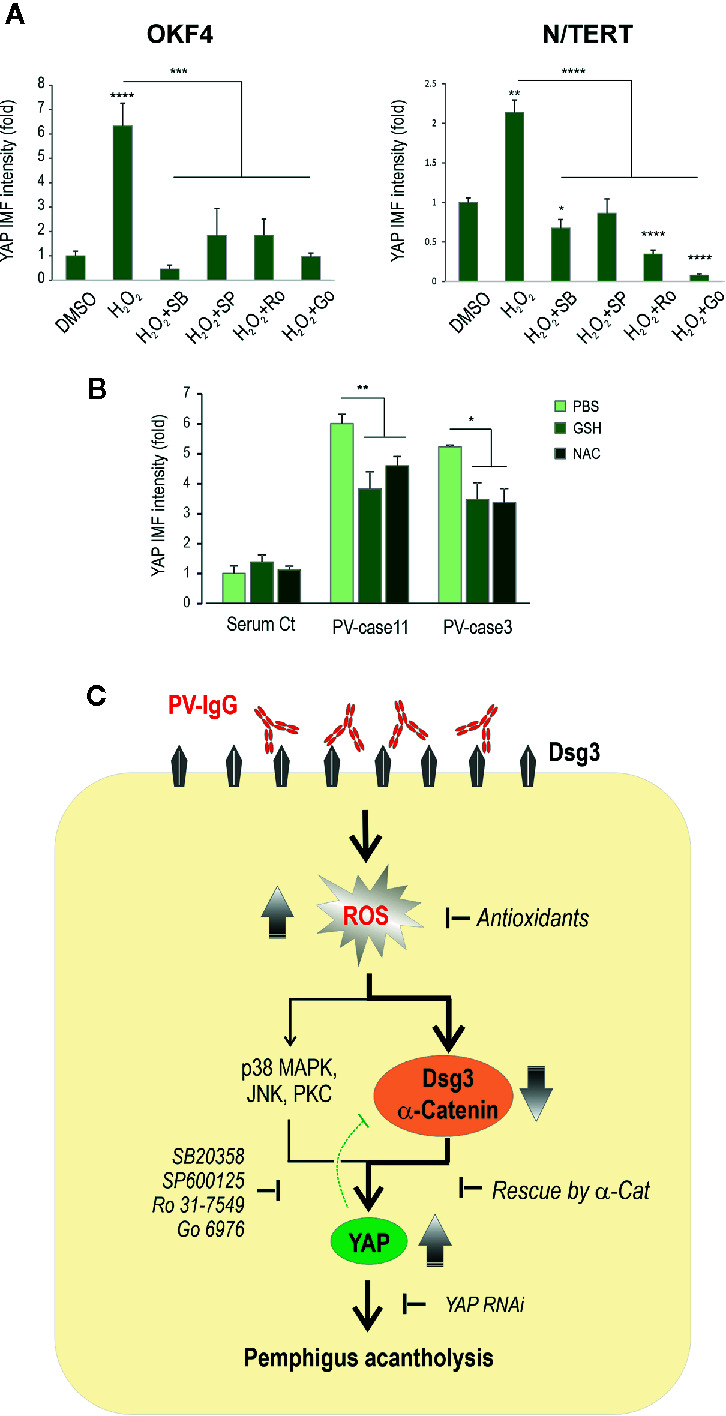
Inhibition of other kinase signaling pathways can also rescue the H_2_O_2_ induced YAP accumulation. **(A)** YAP expression in cells treated with H_2_O_2_ in the presence and absence of various inhibitors for p38MAPK (SB203580 (SB), 20μM), JNK (SP600125 (SP), 20μM) and PKC (Ro 31-7549 (Ro), 2μM and Go6976 (Go), 1μM), respectively. Cells seeded at ~70% confluent densities (*i.e.* 2x10^5^ cells per 24-well) were treated with the inhibitors for 1 hour before addition of H_2_O_2_ and incubated further for 4 hours before fixation and immunostaining for YAP. Both OKF4 and N/TERT cell lines were analyzed by image quantitation for total YAP expression (n=5, representative of 2 experiments, Mean ± SEM, one-way ANOVA was used for statistical analysis, *p < 0.05, **p < 0.01, ***p < 0.001, ****p < 0.0001). The corresponding confocal images of YAP staining are displayed in **Figure S4**. Nuc, nucleus; Cyto, cytoplasm. **(B)** Immunofluorescence analysis of YAP staining in primary skin keratinocytes treated with PV sera (two cases) in the presence and absence of one dose of antioxidants, *i.e.* GSH (10µM) or NAC (2mM) for 24 hours. Cells seeded in 96-wells at ~70% confluence (*i.e.* ~1.2x10^5^ cells per 96-well) in KSFM before being exposed to PV sera with -/+ GSH/NAC in calcium-containing KSFM (Ca^++^ 1.5mM) (n=5~7 fields/sample, representative of 3 experiments in three cell lines, Mean ± SEM, *p < 0.05, **p < 0.01). Both GSH and NAC showed to blunt the PV sera-induced YAP levels to some extent compared to PV sera treated cells. **(C)** Schematic diagram illustrating that PV-IgG targeting Dsg3 causes oxidative stress in keratinocytes with ROS overproduction that, in turn, elicits disruption of cell junction assembly proteins such as Dsg3 and α-catenin, leading to YAP accumulation (dysregulation) and ultimately, pemphigus acantholysis. ROS mediated YAP dysregulation may also be triggered by activations of other signaling pathways such as p38MAPK, JNK and PKC, and inhibition of these pathways can effectively prevent YAP accumulation. The green dotted bar-headed line indicates the negative feedback mechanism of YAP regulation to Dsg3.

Activation of several pathways including p38MAPK, JNK and PKC is triggered by PV-IgG (2, 5, 50, 51). Additionally, other independent studies indicate that ROS can amplify these signaling pathways (52–55). To address whether inhibition of these pathways could block YAP accumulation induced by H_2_O_2_, we performed experiments in N/TERT and OKF4 lines that were subjected to H_2_O_2_ exposure in conjunction with various inhibitors before YAP immunofluorescence. Cells were treated with H_2_O_2_ (200µM) in the presence and absence of inhibitors, *i.e.* SB203580 (20μM) for p38MAPK (37), SP600125 (20μM) for JNK (56), Ro 31-7549 (2μM) and Go6976 (1μM) for PKC (57). Again, they were treated with inhibitors 1 hour before addition of H_2_O_2_ and incubated for additional 4 hours before fixation. Image quantitation indicated marked inhibition of YAP, as well as its nuclear accumulation (especially in N/TERT treated with PKC inhibitors), as opposed to positive control of H_2_O_2_ treated cells, with the levels being comparable to the baseline of vehicle control or even lower (**Figure 8A**, **Figure S4**)

### Antioxidant Treatment of Keratinocytes Suppresses PV Sera-Induced YAP Accumulation

Finally, to test whether antioxidants are able to suppress PV sera-induced YAP accumulation, we treated primary skin keratinocytes with PV sera in conjunction with one dose of GSH (10µM) and NAC (2mM), respectively, for 24 hours before immunostaining for YAP and the resulting data of the image quantitation indicated a suppression of total YAP by either GSH or NAC to some extent (**Figure 8B**).

Collectively, the results in this study suggest that PV-IgG is capable of inducing excessive ROS production in keratinocytes that, in turn, causes depletion of Dsg3 and α-catenin at the cell junctions, resulting in YAP dysregulation with the consequence of pemphigus acantholysis (**Figure 8C**). In line with these findings, knocking down of YAP was capable of abolishing the negative impact of the anti-Dsg3 antibody AK23 on Dsg3 depletion.

## Discussion

This study provides novel evidence of YAP dysregulation in PV provoked by oxidative stress, mediated by PV sera/anti-Dsg3 IgG binding to Dsg3, a recently characterized anti-stress protein (37). In addition, it demonstrated that antioxidants are capable of mitigating such effects by suppressing YAP with concomitant enhancement in cell-cell adhesion. Several lines of evidence are demonstrated here: 1) elevated YAP was detected in oral tissues of PV patients *in vivo* and also recapitulated in oral/skin keratinocytes treated with PV sera and the pathogenic anti-Dsg3 antibody *in vitro*; 2) In parallel, elevated ROS was detected in these PV-IgG treated cells, indicating oxidative stress of keratinocytes evoked by PV-IgG targeting Dsg3; 3) Expression of α-catenin could rescue H_2_O_2_ induced YAP accumulation leading to its nuclear exclusion; 4) Similar effects were found in antioxidants treated cells exposed to H_2_O_2_ or PV sera, with the antioxidants strengthening cell-cell adhesion, suggesting that suppression of oxidative stress can inhibit YAP dysregulation and enhance the intercellular junction integrity; 5) Overexpression of YAP elicited perturbation of junction formation with an inverse effect seen by YAP depletion. In conclusion, these results indicate that dysregulated YAP, coupled with aberrant ROS production, are likely contributors to the pathogenesis of PV (**Figure 8C**).

Given that YAP is increasingly recognized to play a role in various disease processes (23, 58), elucidation of altered YAP in pemphigus is crucial to further our knowledge of PV pathology. In this study, we have identified aberrant accumulation of YAP in the oral mucous membrane of 60% of PV patients. Intriguingly, little YAP was detected in normal tissue suggesting its degradation controlled by active Hippo signaling coupled with the maturation of cell-cell junctions. The heterogeneity of YAP steady-state protein levels in PV patients, including nuclear YAP detection in non-lesional regions, could well reflect variations of clinical activities/treatment status of the disease, surface Dsg3 depletion with different antibody profiles, and/or the dynamic nature of YAP in response to cellular stress. In line with this finding, variations of p53 in PV were also reported in our previous study (37). *In vitro* study demonstrated elevated YAP in N/TERT and primary keratinocytes treated with PV sera and/or AK23 that binds to the extracellular adhesion site of Dsg3. It is worth noting that the majority of PV sera used in this study were collected in the dermatology clinic so it is likely that some sera samples also contained anti-Dsg1 antibodies from patients with a mucocutaneous form of PV. Thus, the possibility of some effect caused by anti-Dsg1 is not excluded from this study. It is possible that disruption of cell junctions by anti-Dsg1 antibodies also evokes YAP alterations due to the adhesive role of Dsg1 in desmosomes, as we also observed a moderate increase of YAP in pemphigus foliaceus but little or no changes in lichen planus, a chronic inflammatory, autoimmune condition in which cell junctions are much less affected (our unpublished data). Some variations in YAP distribution were observed between oral and skin keratinocytes treated with AK23. While elevated YAP was shown in the cytoplasm of N/TERTs, predominant YAP nuclear staining was detected in oral OKF4 cells in AK23 dose-dependent experiment (**Figure 2D**, **Figure S1C**). Nevertheless, pronounced YAP cytoplasmic accumulation was shown consistently in both lines treated with PV sera. Intriguingly, some YAP aggregates were found at the borders of keratinocytes treated with PV sera, especially in cells with the significant loss of Dsg3. However, such a staining pattern was hardly detected in cells treated with AK23, the monospecific antibody against Dsg3 (**Figure 2**), suggesting that the YAP aggregates at the border of PV sera treated cells may somehow be caused by polyclonal antibodies targeting Dsg3 and/or Dsg1.

The biological function of YAP accumulation in PV remains unclear, which may be due to the defect in Hippo-YAP response to tissue stress and disruption of cell junctions, including adhesion molecules Dsg3 and α-catenin, both of which have been identified as the components of epithelial Hippo network and the upstream regulator of YAP (29, 43). E-cadherin mediated complex formation with α-catenin during junction assembly, which also involves Dsg3 (16, 59), is shown to serve as an upstream regulator of Hippo signaling that negatively regulates YAP and its nuclear exclusion (44). In support of this, we show here that the expression of exogenous α-catenin could facilitate cytoplasmic localization of YAP from the nucleus induced by H_2_O_2_. Our recent report has revealed that Dsg3 plays a role in regulating YAP by forming a complex with and recruiting YAP from the nucleus (29). This process requires plakophilins-1/3 that bridge Dsg3 and YAP in junction assembly (29). Cell-cell adhesion is severely perturbed in PV due to anti-Dsg3/Dsg1 antibodies. Thus, the defect in cell cohesion can have an impact on Hippo kinases and this could be a causative factor for YAP stabilization in PV. The Hippo signaling pathway is involved in response to a wide range of extracellular and intracellular signals, including cell-cell contact, cell polarity and mechanical cues (25). Disruption of cell junctions affects cell morphology, polarization and the mechanical properties of epithelia, rendering defective Hippo signaling and therefore YAP stabilization.

The impact of YAP modulation on the intercellular junction assembly was investigated in this study that demonstrated its overexpression evoked perturbation in junction formation (**Figures 7A–D**), implying a likelihood in the changes of some cell proliferation markers such as c-Myc as reported by others (15, 60). In line with this finding, YAP depletion resulted in enhanced expression of Dsg3 and α-catenin as well as desmosomal plaque proteins PKP1/3 (**Figure 7E**, unpublished data) that was able to abolish the pathogenicity of AK23 to Dsg3 (**Figure 7F**), suggesting that PV acantholysis is likely caused by YAP accumulation and dysregulation. These data further confirm the reciprocal negative correlation between YAP and cell-cell adhesion that is in line with Hippo. In addition, YAP regulation can be mediated by other kinases, including JNK, p38MAPK, PKC and SRC family tyrosine kinases independent of Hippo signaling (25). Given that activation of SRC, JNK, p38MAPK and PKC signaling have been independently implicated in PV (10, 13, 16, 18, 19, 61, 62), it is not surprising that YAP dysregulation is detected in PV. Alternatively, this could be due to the YAP response to stress signals caused by disruption of cell junctions since both Dsg3 and YAP have been identified to belong to the cellular stress network (23, 29, 58, 63). Accordantly, the osmotic stress-induced activation of p38MAPK was shown to drive cytoplasmic translocation of YAP as an adaptive response to stress, a process independent of Hippo kinases (63). In support, we demonstrated that inhibition of p38MAPK, JNK and PKC had a significant effect on YAP suppression in both oral and skin keratinocytes, especially with the PKC inhibitors in N/TERT (**Figure 8A**, **Figure S4**). Thus, YAP upregulation in PV is likely caused by both the Hippo-dependent and independent activation of various signaling pathways. Importantly, we also showed that antioxidants such as GSH and NAC were capable of blocking PV sera induced YAP expression in primary keratinocytes (**Figure 8B**).

Our study has also identified oxidative stress evoked by PV-IgG targeting Dsg3. The interplay between YAP and ROS was established here and it described a negative impact of elevated YAP and ROS on Dsg3 and α-catenin. The augmented YAP might provide a protective role to combat oxidative stress and the pre-apoptosis machinery driven by caspase-3 activation shown in PV (37, 64). ROS are ubiquitous and short-lived molecules and low sub-cytotoxic concentrations of ROS function as important physiological signaling molecules at multiple points mediating necessary cellular activities such as cell growth and differentiation. However, overproduction of ROS causes oxidative stress leading to cell death and tissue damage. In this regard, it was not surprising that we observed cell loss in some PV serum treated wells. Oxidative stress is involved in various pathological processes, including DNA damage, proliferation, cell adhesion, and survival. Our recent study uncovers Dsg3 acting as the central hub in a signaling network that controls cell-cell adhesion, polarization, cell fate decision and survival (38). Thus, its disruption by PV-IgG can lead to death or alteration in cell proliferation markers (15, 37, 60). Our recent study has uncovered elevated p53 expression in PV *in vivo* and *in vitro* and p53 is known to be capable of regulating ROS *via* a collection of target genes with strong pro-oxidant properties (65). Thus, the increased ROS detected in PV sera and AK23 treated cells could be due to the enhanced p53 activity. Nonetheless, oxidative stress in PV has only been reported in limited studies (30–33) and so far there is little *in vitro* evidence implicating oxidative stress. Our study provides evidence of transient ROS overproduction induced by PV-IgG. ROS are both downstream as well as upstream signaling molecules to PKC and provide signal amplification in human peritoneal mesothelial cells (52). The effect of oxidant stress on intercellular junctions and cytoskeletal remodeling is not new and has been shown in multiple systems (46, 66). Mechanistically, these processes are involved in the inactivation of protein phosphatases, leading to enhanced activities of kinases in several signaling pathways, *e.g.* SRC, PKC, and Rac. The adherens junction proteins are the primary targets of SRC signaling, resulting in tyrosine phosphorylation of proteins, including α-catenin, leading to disruption of cell junctions with enhanced permeability of cell monolayers (46, 59, 66, 67). Notably, these defects can be blocked by ROS scavenging, with an effect on YAP expression (**Figures 6D, E**). We have previously shown that elevated phospho-SRC is found in PV accompanied by altered E-cadherin at the cell junctions (16). The reduced α-catenin in cells treated with anti-Dsg3 antibody or H_2_O_2_ indicated that oxidative stress provokes disruption of both desmosomes and adherens junctions, highlighting the possibility of oxidative stress being responsible, at least in part, for pemphigus acantholysis. In accord, other studies report that H_2_O_2_ triggers short-term cell-cell dissociation and cytoskeletal remodeling accompanied by temporal cell morphological changes in retinal pigment and gingival epithelial cells with antioxidants being effective to inhibit the ROS mediated reduction of E-cadherin and the epithelial barrier function (45, 46). A recent report indicates p38MAPK inhibition alone is not sufficient in preventing PV-IgG induced mucosal blistering in PV (68). Hence, it is likely that the complex, multiple regulatory mechanisms controlled by oxidative stress are responsible for the pathogenesis of PV, as reported by several studies (2, 5, 50, 51).

Although it could be more complicated in nature, the simplified explanation could well be that the Hippo-dependent mechanism involves Dsg3 and α-catenin and the Hippo-independent process involves activation of the MAPK signaling pathways. Thus, there are two events in our proposed model (**Figure 8C**), *i.e.* oxidative stress evoked by PV-IgG targeting Dsg3 and dysregulated YAP induced by ROS, both of which involves α-catenin that links these two events. Oxidative stress causes a reduction of α-catenin that leads to YAP upregulation with concomitant disruption of cell cohesion. Additionally, YAP accumulation can also, in part, be mediated *via* activation of the MAPK pathways. Dsg3 also plays an essential role here due to its function as a component in the cell anti-stress network beyond cell adhesion. Since both Dsg3 and YAP are the components in this network, they likely are regulated to one another at the gene and protein levels and are sensitive to regulatory signaling in response to various stresses. Further study is necessary to delineate the molecular basis underlying these processes. Our previous study based on loss of Dsg3 function approach suggests that Dsg3 positively regulates YAP (Uttagomol et al., 2019, our unpublished data); paradoxically, increased YAP was detected *via* antibodies binding to Dsg3/1 that also render Dsg3 depletion. However, apparently different mechanisms are involved in these two scenarios, as discussed above. Regarding the YAP/Dsg3 axis, this study provided evidence indicating the negative feedback mechanism, a common principle in signal transduction that controls the expression levels of Dsg3 and other junctional proteins, and this mechanism may play an essential role in maintaining the balance between cell proliferation and differentiation and beyond. In conclusion, the results of this study provide new evidence on oxidative stress and YAP dysregulation that may contribute to the pathogenesis of PV and submits that antioxidants have therapeutic potential for use in the treatment of pemphigus vulgaris, a devastating mucocutaneous blistering disease.

## Data Availability Statement

The original contributions presented in the study are included in the article/**Supplementary Material**, further inquiries can be directed to the corresponding author.

## Author Contributions

HW, YH, and YC designed research. YH, AR, HW, LG, UA, and JU performed experiments and data analysis. YC, HJ, FF, and EP contributed PV specimens/sera/cell line/reagents, respectively. HW wrote the manuscript. HW, EP, FF, and AR edited the manuscript and all authors approved the manuscript. All authors contributed to the article and approved the submitted version.

## Funding

This work was supported by Barts Charity grant (IRMG1A8R). USA was funded by charity Elfarouq Foundation, Suite 201 Stanmore Business & Innovation Centre, Stanmore Place, Howard Road, London HA7 1BT. JU was funded by a scholarship from Naresuan University, Phitsanulok, Thailand.

## Conflict of Interest

The authors declare that the research was conducted in the absence of any commercial or financial relationships that could be construed as a potential conflict of interest.

## References

[1] Amagai M. Autoimmune and infectious skin diseases that target desmogleins. Proc Jpn Acad Ser B Phys Biol Sci (2010) 86:524–37. 10.2183/pjab.86.524 PMC3108298 20467217

[2] Kitajima Y. 150(th) anniversary series: Desmosomes and autoimmune disease, perspective of dynamic desmosome remodeling and its impairments in pemphigus. Cell Commun Adhes (2014) 21:269–80. 10.3109/15419061.2014.943397 25078507

[3] Lanza A Cirillo N Femiano F Gombos F. How does acantholysis occur in pemphigus vulgaris: a critical review. J Cutan Pathol (2006) 33:401–12. 10.1111/j.0303-6987.2006.00523.x 16776715

[4] Waschke J. The desmosome and pemphigus. Histochem Cell Biol (2008) 130:21–54. 10.1007/s00418-008-0420-0 18386043 PMC2413110

[5] Spindler V Eming R Schmidt E Amagai M Grando S Jonkman MF. Mechanisms Causing Loss of Keratinocyte Cohesion in Pemphigus. J Invest Dermatol (2018) 138:32–7. 10.1016/j.jid.2017.06.022 29037765

[6] Heupel WM Zillikens D Drenckhahn D Waschke J. Pemphigus vulgaris IgG directly inhibit desmoglein 3-mediated transinteraction. J Immunol (2008) 181:1825–34. 10.4049/jimmunol.181.3.1825 18641320

[7] Tsunoda K Ota T Aoki M Yamada T Nagai T Nakagawa T. Induction of pemphigus phenotype by a mouse monoclonal antibody against the amino-terminal adhesive interface of desmoglein 3. J Immunol (2003) 170:2170–8. 10.4049/jimmunol.170.4.2170 12574390

[8] Amagai M Karpati S Prussick R Klaus-Kovtun V Stanley JR. Autoantibodies against the amino-terminal cadherin-like binding domain of pemphigus vulgaris antigen are pathogenic. J Clin Invest (1992) 90:919–26. 10.1172/JCI115968 PMC329947 1522242

[9] Caldelari R de Bruin A Baumann D Suter MM Bierkamp C Balmer V. A central role for the armadillo protein plakoglobin in the autoimmune disease pemphigus vulgaris. J Cell Biol (2001) 153:823–34. 10.1083/jcb.153.4.823 PMC2192383 11352942

[10] Kawasaki Y Aoyama Y Tsunoda K Amagai M Kitajima Y. Pathogenic monoclonal antibody against desmoglein 3 augments desmoglein 3 and p38 MAPK phosphorylation in human squamous carcinoma cell line. Autoimmunity (2006) 39:587–90. 10.1080/08916930600971943 17101502

[11] Berkowitz P Hu P Liu Z Diaz LA Enghild JJ Chua MP. Desmosome signaling. Inhibition of p38MAPK prevents pemphigus vulgaris IgG-induced cytoskeleton reorganization. J Biol Chem (2005) 280:23778–84. 10.1074/jbc.M501365200 15840580

[12] Vielmuth F Waschke J Spindler V. Loss of Desmoglein Binding Is Not Sufficient for Keratinocyte Dissociation in Pemphigus. J Invest Dermatol (2015) 135:3068–77. 10.1038/jid.2015.324 26288352

[13] Mao X Sano Y Park JM Payne AS. p38 MAPK activation is downstream of the loss of intercellular adhesion in pemphigus vulgaris. J Biol Chem (2011) 286:1283–91. 10.1074/jbc.M110.172874 PMC3020736 21078676

[14] Spindler V Waschke J. Role of Rho GTPases in desmosomal adhesion and pemphigus pathogenesis. Ann Anat (2011) 193:177–80. 10.1016/j.aanat.2011.02.003 21441018

[15] Williamson L Raess NA Caldelari R Zakher A de Bruin A Posthaus H. Pemphigus vulgaris identifies plakoglobin as key suppressor of c-Myc in the skin. EMBO J (2006) 25:3298–309. 10.1038/sj.emboj.7601224 PMC1523185 16871158

[16] Tsang SM Brown L Lin K Liu L Piper K O’Toole EA. Non-junctional human desmoglein 3 acts as an upstream regulator of Src in E-cadherin adhesion, a pathway possibly involved in the pathogenesis of pemphigus vulgaris. J Pathol (2012) 227:81–93. 10.1002/path.3982 22294297

[17] Chernyavsky AI Arredondo J Kitajima Y Sato-Nagai M Grando SA. Desmoglein versus non-desmoglein signaling in pemphigus acantholysis: characterization of novel signaling pathways downstream of pemphigus vulgaris antigens. J Biol Chem (2007) 282:13804–12. 10.1074/jbc.M611365200 17344213

[18] Marchenko S Chernyavsky AI Arredondo J Gindi V Grando SA. Antimitochondrial autoantibodies in pemphigus vulgaris: a missing link in disease pathophysiology. J Biol Chem (2010) 285:3695–704. 10.1074/jbc.M109.081570 PMC2823510 20007702

[19] Grando SA. Pemphigus autoimmunity: hypotheses and realities. Autoimmunity (2012) 45:7–35. 10.3109/08916934.2011.606444 21939410 PMC3251002

[20] Sinha AA Sajda T. The Evolving Story of Autoantibodies in Pemphigus Vulgaris: Development of the “Super Compensation Hypothesis”. Front Med (Lausanne) (2018) 5:218. 10.3389/fmed.2018.00218 30155465 PMC6102394

[21] Nguyen VT Ndoye A Shultz LD Pittelkow MR Grando SA. Antibodies against keratinocyte antigens other than desmogleins 1 and 3 can induce pemphigus vulgaris-like lesions. J Clin Invest (2000) 106:1467–79. 10.1172/JCI10305 PMC387253 11120754

[22] Zhu C Li L Zhao B. The regulation and function of YAP transcription co-activator. Acta Biochim Biophys Sin (Shanghai) (2015) 47:16–28. 10.1093/abbs/gmu110 25487920

[23] Piccolo S Dupont S Cordenonsi M. The biology of YAP/TAZ: hippo signaling and beyond. Physiol Rev (2014) 94:1287–312. 10.1152/physrev.00005.2014 25287865

[24] Aqeilan RI. Hippo signaling: to die or not to die. Cell Death Differ (2013) 20:1287–8. 10.1038/cdd.2013.100 PMC3770324 24013777

[25] Yu FX Zhao B Guan KL. Hippo Pathway in Organ Size Control, Tissue Homeostasis, and Cancer. Cell (2015) 163:811–28. 10.1016/j.cell.2015.10.044 PMC4638384 26544935

[26] Shao D Zhai P Del Re DP Sciarretta S Yabuta N Nojima H. A functional interaction between Hippo-YAP signalling and FoxO1 mediates the oxidative stress response. Nat Commun (2014) 5:3315. 10.1038/ncomms4315 24525530 PMC3962829

[27] Chen SN Gurha P Lombardi R Ruggiero A Willerson JT Marian AJ. The hippo pathway is activated and is a causal mechanism for adipogenesis in arrhythmogenic cardiomyopathy. Circ Res (2014) 114:454–68. 10.1161/CIRCRESAHA.114.302810 PMC3946717 24276085

[28] Plouffe SW Hong AW Guan KL. Disease implications of the Hippo/YAP pathway. Trends Mol Med (2015) 21:212–22. 10.1016/j.molmed.2015.01.003 PMC4385444 25702974

[29] Uttagomol J Ahmad US Rehman A Huang Y Laly AC Kang A. Evidence for the Desmosomal Cadherin Desmoglein-3 in Regulating YAP and Phospho-YAP in Keratinocyte Responses to Mechanical Forces. Int J Mol Sci (2019) 20(24):6221. 10.1101/827725 PMC6940936 31835537

[30] Shah AA Dey-Rao R Seiffert-Sinha K Sinha AA. Increased oxidative stress in pemphigus vulgaris is related to disease activity and HLA-association. Autoimmunity (2016) 49:248–57. 10.3109/08916934.2016.1145675 26911801

[31] Javanbakht MH Djalali M Daneshpazhooh M Zarei M Eshraghian MR Derakhshanian H. Evaluation of antioxidant enzyme activity and antioxidant capacity in patients with newly diagnosed pemphigus vulgaris. Clin Exp Dermatol (2015) 40:313–7. 10.1111/ced.12489 25683954

[32] Yesilova Y Ucmak D Selek S Dertlioglu SB Sula B Bozkus F. Oxidative stress index may play a key role in patients with pemphigus vulgaris. J Eur Acad Dermatol Venereol (2013) 27:465–7. 10.1111/j.1468-3083.2012.04463.x 22324759

[33] Abida O Gargouri B Ben MR Mseddi-Djemal M Masmoudi A Ben AM. Biomarkers of oxidative stress in epidermis of Tunisian pemphigus foliaceus patients. J Eur Acad Dermatol Venereol (2013) 27:e271–5. 10.1111/j.1468-3083.2012.04626.x 22738420

[34] Mao B Gao Y Bai Y Yuan Z. Hippo signaling in stress response and homeostasis maintenance. Acta Biochim Biophys Sin (Shanghai) (2015) 47:2–9. 10.1093/abbs/gmu109 25476206

[35] Dixit D Ghildiyal R Anto NP Sen E. Chaetocin-induced ROS-mediated apoptosis involves ATM-YAP1 axis and JNK-dependent inhibition of glucose metabolism. Cell Death Dis (2014) 5:e1212. 10.1038/cddis.2014.179 24810048 PMC4047915

[36] Roh KH Choi EJ. TRAF2 functions as an activator switch in the reactive oxygen species-induced stimulation of MST1. Free Radic Biol Med (2016) 91:105–13. 10.1016/j.freeradbiomed.2015.12.010 26698664

[37] Rehman A Cai Y Hunefeld C Jedlickova H Huang Y Teck TM. The desmosomal cadherin desmoglein-3 acts as a keratinocyte anti-stress protein via suppression of p53. Cell Death Dis (2019) 10:750. 10.1038/s41419-019-1988-0 31582719 PMC6776551

[38] Li X Ahmad US Huang Y Uttagomol J Rehman A Zhou K. Desmoglein-3 acts as a pro-survival protein by suppressing reactive oxygen species and doming whilst augmenting the tight junctions in MDCK cells. Mech Ageing Dev (2019) 184:111174. 10.1016/j.mad.2019.111174 31678215

[39] Rheinwald JG Hahn WC Ramsey MR Wu JY Guo Z Tsao H. A two-stage, p16(INK4A)- and p53-dependent keratinocyte senescence mechanism that limits replicative potential independent of telomere status. Mol Cell Biol (2002) 22:5157–72. 10.1128/MCB.22.14.5157-5172.2002 PMC139780 12077343

[40] Dickson MA Hahn WC Ino Y Ronfard V Wu JY Weinberg RA. Human keratinocytes that express hTERT and also bypass a p16(INK4a)-enforced mechanism that limits life span become immortal yet retain normal growth and differentiation characteristics. Mol Cell Biol (2000) 20:1436–47. 10.1128/MCB.20.4.1436-1447.2000 PMC85304 10648628

[41] Moftah H Dias K Apu EH Liu L Uttagomol J Bergmeier L. Desmoglein 3 regulates membrane trafficking of cadherins, an implication in cell-cell adhesion. Cell Adh Migr (2016) 11(3):211–32. 10.1080/19336918.2016.1195942 PMC5479455 27254775

[42] Jennings JM Tucker DK Kottke MD Saito M Delva E Hanakawa Y. Desmosome disassembly in response to pemphigus vulgaris IgG occurs in distinct phases and can be reversed by expression of exogenous dsg3. J Invest Dermatol (2011) 131:706–18. 10.1038/jid.2010.389 PMC3235416 21160493

[43] Schlegelmilch K Mohseni M Kirak O Pruszak J Rodriguez JR Zhou D. Yap1 acts downstream of alpha-catenin to control epidermal proliferation. Cell (2011) 144:782–95. 10.1016/j.cell.2011.02.031 PMC3237196 21376238

[44] Kim NG Koh E Chen X Gumbiner BM. E-cadherin mediates contact inhibition of proliferation through Hippo signaling-pathway components. Proc Natl Acad Sci U S A (2011) 108:11930–5. 10.1073/pnas.1103345108 PMC3141988 21730131

[45] Abe-Yutori M Chikazawa T Shibasaki K Murakami S. Decreased expression of E-cadherin by Porphyromonas gingivalis-lipopolysaccharide attenuates epithelial barrier function. J Periodontal Res (2017) 52:42–50. 10.1111/jre.12367 27016120

[46] Inumaru J Nagano O Takahashi E Ishimoto T Nakamura S Suzuki Y. Molecular mechanisms regulating dissociation of cell-cell junction of epithelial cells by oxidative stress. Genes Cells (2009) 14:703–16. 10.1111/j.1365-2443.2009.01303.x 19422420

[47] Kim KA Jung JH Kang IG Choi YS Kim ST. ROS Is Involved in Disruption of Tight Junctions of Human Nasal Epithelial Cells Induced by HRV16. Laryngoscope (2018) 128:E393–401. 10.1002/lary.27510 30325507

[48] Narimatsu T Ozawa Y Miyake S Kubota S Hirasawa M Nagai N. Disruption of cell-cell junctions and induction of pathological cytokines in the retinal pigment epithelium of light-exposed mice. Invest Ophthalmol Vis Sci (2013) 54:4555–62. 10.1167/iovs.12-11572 23761083

[49] Vasioukhin V Bauer C Degenstein L Wise B Fuchs E. Hyperproliferation and defects in epithelial polarity upon conditional ablation of alpha-catenin in skin. Cell (2001) 104:605–17. 10.1016/S0092-8674(01)00246-X 11239416

[50] Wan H. Desmoglein-3. In: Choi S, editor. Encyclopedia of Signaling Molecules. New York, NY: Springer New York (2016). p. 1–15. 10.1007/978-1-4614-6438-9_101583-1

[51] Ahmed AR Carrozzo M Caux F Cirillo N Dmochowski M Alonso AE. Monopathogenic vs multipathogenic explanations of pemphigus pathophysiology. Exp Dermatol (2016) 25:839–46. 10.1111/exd.13106 27305362

[52] Lee HB Yu MR Song JS Ha H. Reactive oxygen species amplify protein kinase C signaling in high glucose-induced fibronectin expression by human peritoneal mesothelial cells. Kidney Int (2004) 65:1170–9. 10.1111/j.1523-1755.2004.00491.x 15086456

[53] Sato A Okada M Shibuya K Watanabe E Seino S Narita Y. Pivotal role for ROS activation of p38 MAPK in the control of differentiation and tumor-initiating capacity of glioma-initiating cells. Stem Cell Res (2014) 12:119–31. 10.1016/j.scr.2013.09.012 24185179

[54] Son Y Cheong YK Kim NH Chung HT Kang DG Pae HO. Mitogen-Activated Protein Kinases and Reactive Oxygen Species: How Can ROS Activate MAPK Pathways? J Signal Transduction (2011) 2011:792639. 10.1155/2011/792639 PMC3100083 21637379

[55] Son Y Kim S Chung HT Pae HO. Reactive oxygen species in the activation of MAP kinases. Methods Enzymol (2013) 528:27–48. 10.1016/B978-0-12-405881-1.00002-1 23849857

[56] Wang Y Bell JC Keeney DS Strobel HW. Gene regulation of CYP4F11 in human keratinocyte HaCaT cells. Drug Metab Dispos (2010) 38:100–7. 10.1124/dmd.109.029025 PMC2802424 19812349

[57] Brown L Waseem A Cruz IN Szary J Gunic E Mannan T. Desmoglein 3 promotes cancer cell migration and invasion by regulating activator protein 1 and protein kinase C-dependent-Ezrin activation. Oncogene (2014) 33:2363–74. 10.1038/onc.2013.186 23752190

[58] Panciera T Azzolin L Cordenonsi M Piccolo S. Mechanobiology of YAP and TAZ in physiology and disease. Nat Rev Mol Cell Biol (2017) 18:758–70. 10.1038/nrm.2017.87 PMC6192510 28951564

[59] Tsang SM Liu L Teh MT Wheeler A Grose R Hart IR. Desmoglein 3, via an interaction with E-cadherin, is associated with activation of Src. PloS One (2010) 5:e14211. 10.1371/journal.pone.0014211 21151980 PMC2997060

[60] Williamson L Suter MM Olivry T Wyder M Muller EJ. Upregulation of c-Myc may contribute to the pathogenesis of canine pemphigus vulgaris. Vet Dermatol (2007) 18:12–7. 10.1111/j.1365-3164.2007.00561.x 17222234

[61] Berkowitz P Chua M Liu Z Diaz LA Rubenstein DS. Autoantibodies in the autoimmune disease pemphigus foliaceus induce blistering via p38 mitogen-activated protein kinase-dependent signaling in the skin. Am J Pathol (2008) 173:1628–36. 10.2353/ajpath.2008.080391 PMC2626375 18988808

[62] Jolly PS Berkowitz P Bektas M Lee HE Chua M Diaz LA. p38MAPK signaling and desmoglein-3 internalization are linked events in pemphigus acantholysis. J Biol Chem (2010) 19(285):8936–41. 10.1074/jbc.M109.087999 PMC2838315 20093368

[63] Lin KC Moroishi T Meng Z Jeong HS Plouffe SW Sekido Y. Regulation of Hippo pathway transcription factor TEAD by p38 MAPK-induced cytoplasmic translocation. Nat Cell Biol (2017) 19:996–1002. 10.1038/ncb3581 28752853 PMC5541894

[64] Takaguri A Kubo T Mori M Satoh K. The protective role of YAP1 on ER stress-induced cell death in vascular smooth muscle cells. Eur J Pharmacol (2017) 815:470–7. 10.1016/j.ejphar.2017.09.033 28951205

[65] Budanov AV. The role of tumor suppressor p53 in the antioxidant defense and metabolism. Subcell Biochem (2014) 85:337–58. 10.1007/978-94-017-9211-0_18 PMC4206257 25201203

[66] van WS van Buul JD Quik S Mul FP Anthony EC ten Klooster JP. Reactive oxygen species mediate Rac-induced loss of cell-cell adhesion in primary human endothelial cells. J Cell Sci (2002) 115:1837–46. 10.1242/jcs.115.9.1837 11956315

[67] Lum H Roebuck KA. Oxidant stress and endothelial cell dysfunction. Am J Physiol Cell Physiol (2001) 280:C719–41. 10.1152/ajpcell.2001.280.4.C719 11245588

[68] Egu DT Sigmund AM Schmidt E Spindler V Walter E Waschke J. A new exÂ vivo human oral mucosa model reveals that p38MAPK inhibition is not effective in preventing autoantibody-induced mucosal blistering in pemphigus. Br J Dermatol (2020) 182:987–94. 10.1111/bjd.18237 31218663

