# Supplementary material for: Oxidative Stress-Mediated YAP Dysregulation Contributes to the Pathogenesis of Pemphigus Vulgaris
Source: Front Immunol. 2021 Apr 19;12:649502. doi: 10.3389/fimmu.2021.649502 (PMC8098436; doi:10.3389/fimmu.2021.649502)
Supplement: Supplementary file 1 [file DataSheet_1.pdf]

Supplementary Material

1 Supplementary Data

Materials and Methods

Cell lines, PV sera and clinical patient oral mucosal samples

Two immortalised keratinocyte cell lines. *i.e*. OKF4/CDK4R/P53DD/TERT (OKF4) derived from floor of the mouth and N/TERT derived from skin were used in the study. Oral tissue samples of PV patients (25 PV cases and 10 normal healthy tissue controls as well as 3 cancer patient samples) were obtained from our collaborator in Guiyang Medical University, China and PV sera (anonymous, 10 cases) were received from our collaborator based in First Department of Dermatovenerology, St. Anne's Faculty Hospital, Brno, Czech Republic; all with informed patient consent and local ethical approval by the Insitituional Ethics Committee of the St. Anne's Faculty Hospital and Affiliated Hospital of Guizhou Medical University, respectively. The pathogenic activities of PV sera to induce a significant reduction of Dsg3 in cultured keratinocytes was characterised in our recent report that showed convincingly the depletion of Dsg3 at the cell surface of N/TERT keratinocytes treated with PV sera compared to control serum treated cells (Rehman et al., 2019).

Keratinocyte culture and treatment

Both cell lines were maintained in keratinocyte serum-free medium (KSFM) (17005042, Thermo Scientific) (Rheinwald et al., 2002) and for all experiments, they were cultured in complete keratinocyte growth medium (KGM) containing Dulbecco's Modified Eagle Medium (DMEM) (12–604F, Lonza): Ham's F-12 (11765054, Thermo Scientific) in the ratio of 3:1 supplemented with 10% fetal bovine serum (FBS) (10270106, Gibco), 10 ng/ml epidermal growth factor (EGF) (13247-051, Invitrogen), 5 μg/ml insulin human solution (19278, Sigma), 10^{-10} cholera toxin (C8052, Sigma) and 0.4 μg/ml hydrocortisone (H4001, Sigma) (Rehman et al., 2019). Cells were treated with PV sera (40% in KGM) (Rehman et al., 2019) for 24 hours unless stated otherwise or the mouse monoclonal antibody AK23 at the elevated concentrations in KGM or for various time frames up to 24 hours. For hydrogen peroxide (H_2O_2) treatment, cells were treated with H_2O_2 at various concentrations for 2 hours before fixation and immunofluorescent staining or treatment with CellRox Oxidative Stress Reagent for the measurement of ROS levels in live cells. For antioxidant experiments, cells were pre-treated in the presence and absence of antioxidants such as N-acetyl cysteine (NAC) (2 mM) and glutathione (GSH) (10μM) for 1 hour before addition of H_2O_2. For inhibitor experiments, cells were pre-treated with various inhibitors, such as SB203580 (20 μM, for p38MAPK), SP600125 (20 μM, for JNK), Ro 31-7549 (2 μM, for PKC) and Go6976 (1 μM, for PKCα) for 1 hour before addition of H_2O_2. In both cases, cells were treated for 4 hours before fixation and immunostaining for YAP. In these experiments, cells were either seeded on coverslips in 24-well plates or plated in 96-well plates according to the individual experiments.

Antibodies

The following mouse and rabbit monoclonal/polyclonal antibodies (Abs) were used: D8H1X, rabbit Ab to YAP (D8H1X-XP, Cell Signaling Technology); 5H10, mouse Ab against the N-terminus of

Dsg3 (sc-23912, Santa Cruz); H-145, rabbit Ab against the C-terminus of Dsg3 (sc-20116, Santa Cruz); rabbit Ab to α-catenin (ab2981, Abcam); GFP mouse mAb (Cell Signaling); purified mouse IgG1, k isotype Ctrl antibody (401401, Biolegend); Secondary Abs were Alexa Fluor 488 goat anti-mouse/rabbit IgG (A11029/A11034; Invitrogen) and Alexa Fluor 568 goat anti-mouse/rabbit IgG (A11031/A11036; Thermo Fisher Scientific, UK).

Immunohistochemistry in PV specimens

Oral tissue samples from 25 PV patients and 6 healthy individuals, as well as 3 oral cancer patients, were analyzed by immunohistochemistry with rabbit anti-YAP antibody (ab52771, Abcam). Paraffin-embedded tissue sections were de-paraffinized, hydrated, and heated in EDTA based antigen retrieval solution (pH=8). After washing 3 times in PBS, tissue sections were subjected to incubation in 3% H_2O_2 in PBS for 10 minutes at room temperature (RT) followed by three washes in PBS. Then slides were incubated with primary antibody for YAP (1:100 dilution) at 4°C overnight. The next day, sections were incubated for an additional 30 minutes at 37°C before washing 3x followed by incubation with secondary antibody (PV-6001, Zsbio, China) for 30 minutes at 37°C. Finally, the antibody binding was detected by incubating the tissue slides in a solution of DAB before mounting. Immunochemical positivity was evaluated by two independent pathologists using scoring criteria. Each section was scored by counting the positive among 100 cells per field with a high-power objective; 5 arbitrary fields were selected for scoring. According to the percentage ranges of positivity, the frequencies of expression were categorized into five grades; 0: <5% positive cells; 1: 5~25% positive cells; 2: 25~50% positive cells; 3: 50~75% positive cells; 4: >75% positive cells. The staining intensity was categorized into four grades; 0: nil staining, 1: weak yellow, 2: yellowish-brown, and 3: brown staining. The final score was calculated based on the two categories and scores 0-1 were defined as negative and scores ≥2 were defined as positive, a classical method in pathology score.

Measurement of reactive oxygen species

The cellular reactive oxygen species (ROS) levels were determined by incubating cells in 96-well plate with the CellROX Oxidative Stress Reagents (Molecular Probe by Life Technologies), at a final concentration of 5 µM, for 30 minutes at 37°C before image acquisition with an IN Cell Analyzer 2200 (GE Healthcare, UK). The reagents are membrane-permeable and are non-fluorescent or weakly fluorescent while in a reduced state. Upon oxidation, they change to fluorescent dye exhibiting strong fluorogenic signals that are detectable in live cells by a fluorescence microscope (Li et al., 2019).

Immunofluorescence (IMF) and image analysis

IMF was performed in cells seeded either on coverslips or 96-well plates. Cells were fixed with 3.6% formaldehyde for 10 minutes, followed by treatment with 0.1% Triton X–100 in PBS for 5 minutes. The nonspecific binding sites were blocked for 15-30 minutes with 10% goat serum (Sigma) before the primary and then the secondary antibody incubations, each lasting for 1 hour at RT. Cells were washed 3 times with PBS containing 0.2% Tween 20 after each antibody incubation. Then, coverslips were counterstained with DAPI for 8–10 minutes before a final wash and mounted on slides. For 96-well plates, cells were subjected to counterstain with DAPI and HCS Cellmask Deep Red stain (H32721, Invitrogen) for 120 minutes before PBS washing three times and then image acquisition with a 20x objective in an INCA 2200 imaging system (GE) and 16-25 images were acquired per well in general. Nuclei and fluorescence intensities for each antibody staining were determined using the Developer Toolbox (GE). For coverslips, 5-6 images were acquired with a 40x objective in Leica

DM4000 Epi-Fluorescence microscope or a 63x oil objective in Zeiss 710 Laser Scanning Confocal Microscope. Image quantitation was performed with ImageJ. For analysis of cytoplasmic and nuclear IMF signals, each image was measured before and after being subtracted by the binary image of the DAPI channel for total and cytoplasmic signals, respectively. The nuclear signals were calculated by subtracting the cytoplasmic signals from the total IMF value for each image in an Excel spreadsheet. Finally, the mean IMF intensity per cell for each image was calculated by dividing the total or cytoplasm/nuclear signals, with the cell number before statistical analysis. Data were presented as the mean IMF intensities for total, the cytoplasmic and nuclear ratio in each condition in the graphs.

Plasmid transfection

Briefly, $2x10^5$ cells were seeded into a 6-well plate overnight before being transfected with α-catenin-GFP expression vector using FuGENE HD transfection reagent (Promega). Cells were harvested with 0.25% trypsin/EDTA (T3924, Sigma) the next day and seeded onto coverslips in 24-well plates at densities of approximately 70% confluence overnight before being treated with hydrogen peroxide as described above.

Dispase dissociation assay

The cell-cell adhesive strength in both N/TERT and OKF4 cell populations subjected to hydrogen peroxide treatments at various concentrations or in the presence and absence of antioxidant regents was analyzed by the dispase dissociation assay (Moftah et al., 2016). Briefly, cells were seeded at equal confluent densities in 6-well plates and incubated in KGM. Once they reached confluence after 2~3 days, cells were washed with PBS twice before treatment in 2 ml PBS containing dispase II (2.4 U/ml, Invitrogen) for approximately 20 minutes at 37°C, to release the epithelial sheets from the substrate (Supplementary Figure. S2a). Then, the epithelial sheets were carefully washed with PBS a couple times before being subjected to mechanical stress by pipetting 3 to 5 times with a 1 ml pipette. Fragments in each well were imaged with ChemiDoc MP Imaging System (Bio-Rad) with Image Lab 5.2.1 software. Finally, the number of fragments for each sample was determined with ImageJ software. At least three independent experiments were performed in each cell line and the pooled data were used for statistical analysis.

Statistical Analysis

Experiments were repeated at least three times and the results are presented as mean±SEM unless stated otherwise. To quantify the IMF intensity of the microscopic images, more than 300 cells in each sample were analyzed. Normality of data distribution was checked with GraphPad software before One-way analysis of variance (ANOVA) in multiple group comparisons ($\alpha=0.05$). P values of less than 0.05 was considered statistically significant, *i.e.* *$p<0.05$, **$p<0.01$, ***$p<0.001$, and ****$p<0.0001$. Wherever possible, the comparison between control and test groups was normalized against the control (set as 1) and expressed as a fold change. For comparison between PV patient samples and healthy controls, we used the Chi-Square Test to obtain the p-value.

2 Supplementary Figures

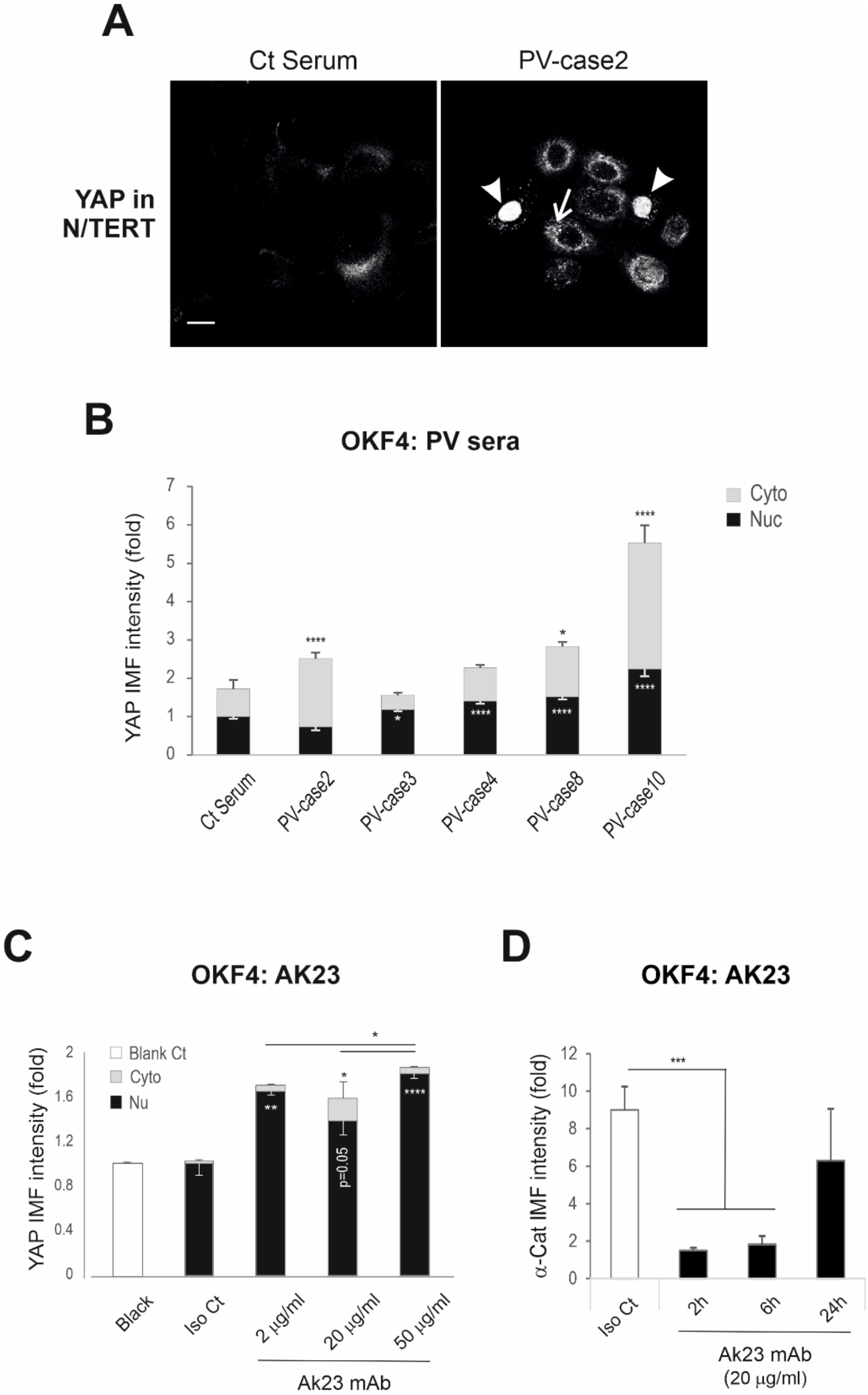

Supplementary Figure 1. Treatment of oral keratinocytes with PV sera or anti-Dsg3 specific antibody results in increased YAP expression. (**A**) Confocal microscopy of N/TERT cells with YAP staining. Cells were seeded at colony density on coverslips and treated with PV serum for 1 day before being

fixed and stained for YAP. Nuclear YAP was detected in cells treated with PV serum (arrowheads) but was not seen in colonies in control-serum treated cells. Arrow indicated YAP cytoplasmic aggregates in PV serum treated cells. (**B**) OKF4 cells seeded in 96-well plate were treated with PV sera (of ten cases together) (40%) for 1 day before fixation and YAP staining. Among 10 wells exposed to PV sera, five wells showed devoid of cells. Images were acquired in the remaining wells with cells still attached with an INCA 2200 (n=25 images per well). The bar chart showed the result of image quantitation. (**C**) OKF4 cells seeded on coverslips were treated with AK23 at the indicated elevated concentrations for 24 hours before YAP staining (n=3~6). (d) Image quantitation for α-catenin staining in cells treated with AK23 for different time points that indicated a significant reduction within 6 hours. (mean±SEM, one-way ANOVA was used for statistical analysis to obtained the p values, *i.e.* *$p<0.05$, **$p<0.01$, ****$p<0.0001$). Data were representative of three independent experiments. Scale bars, 10 µm.

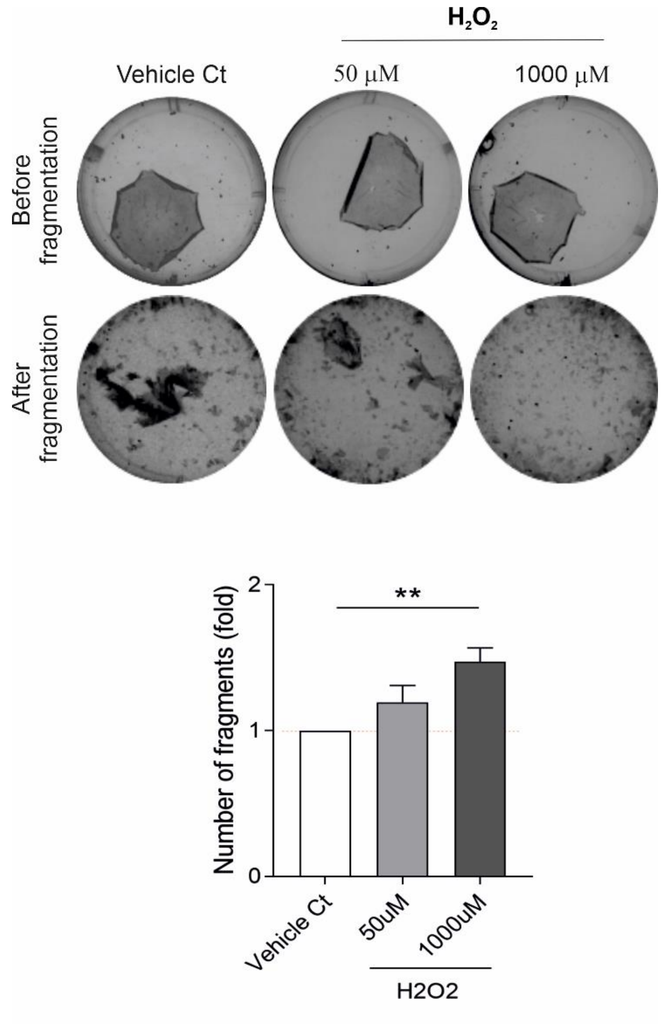

Supplementary Figure 2. Dispase dissociation assay of N/TERTs treated with hydrogen peroxide. Images of epithelial sheets before and after mechanical stress that induced fragmentation are displayed at the top. Cells were seeded at confluent density in 6-well plate and grown for 2~3 days before being treated with H_2O_2 at different concentrations for 4 hours. The medium was replaced with dispase at 2.4 units/ml in PBS that released epithelial sheet from the substrate before fragmentation. Quantitation of the fragments in different conditions was shown below (mean±SEM, data were pooled from at least three independent experiments, one-way ANOVA was used for statistical analysis, **$p<0.01$).

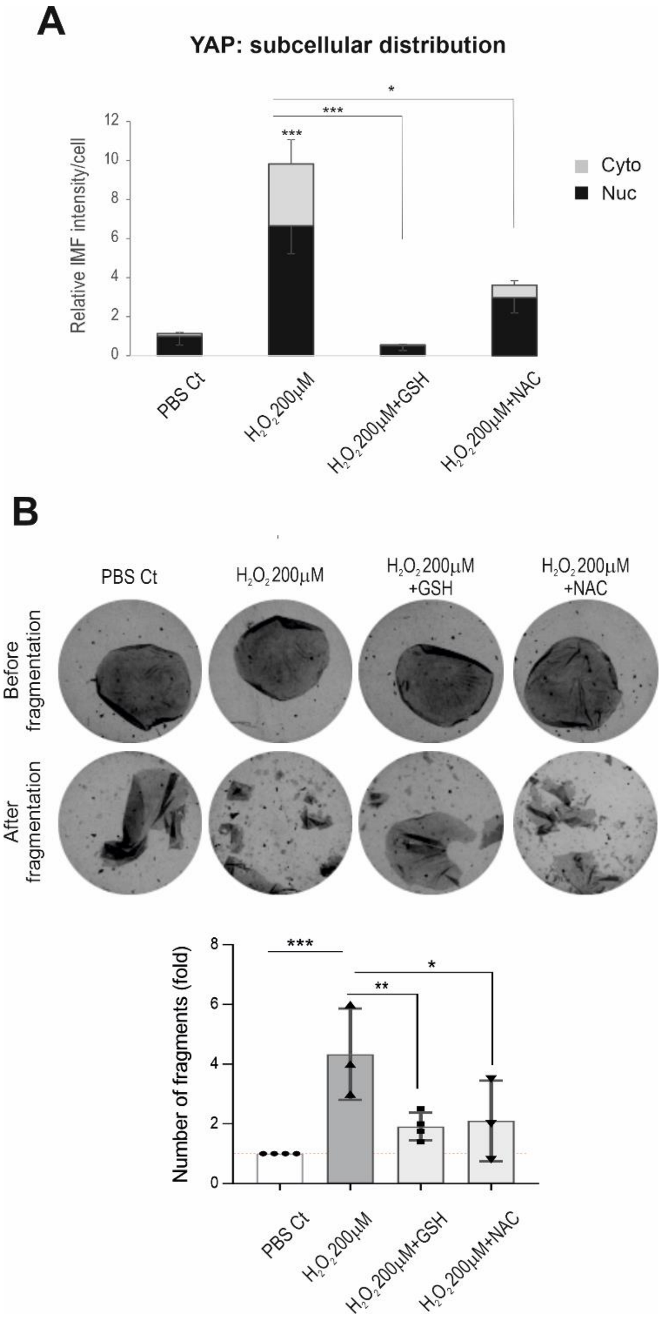

Supplementary Figure 3. Antioxidants were capable of blocking hydrogen peroxide-induced YAP expression and disruption of cell junctional integrity in oral keratinocytes. (**A**) Quantification of YAP staining in OKF4 cells treated with H_2O_2 in the presence and absence of antioxidants GSH (10 µM) and NAC (2 mM). Cells were seeded on coverslips and treated with GSH or NAC for 1 hour before addition of $H_2O_2$and incubated for 2 hours. (**B**) Dispase dissociation assay in OKF4 cells treated with antioxidants as described in a, but in this case, the treatment was for 4 hours rather than 2 hours. Cells were grown in 6-well plate for 2~3 days before being treated with H_2O_2 and/or antioxidants. Data were pooled from four independent experiments. (mean±SD, one-way ANOVA was used for statistical analysis, $^*p<0.05$, $^{**}p<0.01$, $^{***}p<0.001$).

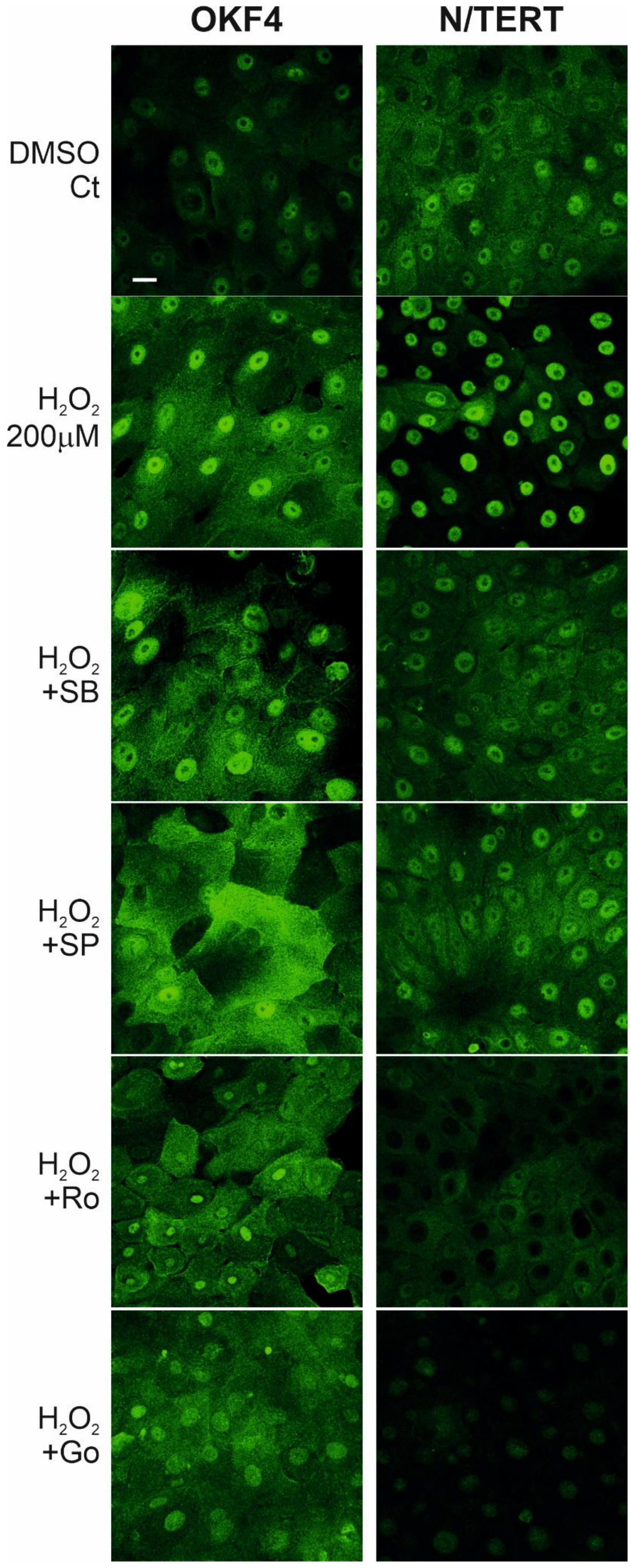

OKF4
N/TERT
DMSO Ct
H2O2 200μM
H2O2 +SB
H2O2 +SP
H2O2 +Ro
H2O2 +Go

Supplementary Figure 4. Confocal microscopy of YAP staining in OKF4 and N/TERT cells treated with various inhibitors. The image quantification data was shown in Figure 6b. Enhanced YAP nuclear staining was evident in H_2O_2 treated cells but this was mitigated in cells treated with inhibitors for p38MAPK, JNK and PKC signal molecules, *i.e.* SB203580 (SB) (for p38MAPK), 20 μM; SP600125 (SP) (for JNK), 20 μM; Ro 31-7549 (Ro), 2 μM and Go6976 (Go), 1 μM (for PKC), respectively. Some different staining patterns were observed in the two cell lines; while OKF4 treated with SP600125 showed predominantly YAP nuclear exclusion, N/TERT displayed nuclear YAP with decreased signals compared to respective H_2O_2 treated cells. OKF4 treated with PKC inhibitors, especially with Ro 31-7549 showed condensed nuclei and diffuse cytoplasmic YAP but this was not seen in N/TERTs in which YAP signal was remarkably reduced. Scale bars, 10μm.

References

Li, X., Ahmad, U. S., Huang, Y., Uttagomol, J., Rehman, A., Zhou, K. et al. (2019). Desmoglein-3 acts as a pro-survival protein by suppressing reactive oxygen species and doming whilst augmenting the tight junctions in MDCK cells. Mech Ageing Dev. 184, 111174.

Moftah, H., Dias, K., Apu, E. H., Liu, L., Uttagomol, J., Bergmeier, L. et al. (2016). Desmoglein 3 regulates membrane trafficking of cadherins, an implication in cell-cell adhesion. Cell Adh Migr. 1-22.

Rehman, A., Cai, Y., Hunefeld, C., Jedlickova, H., Huang, Y., Teck, T. M. et al. (2019). The desmosomal cadherin desmoglein-3 acts as a keratinocyte anti-stress protein via suppression of p53. Cell Death Dis. 10, 750.

Rheinwald, J. G., Hahn, W. C., Ramsey, M. R., Wu, J. Y., Guo, Z., Tsao, H. et al. (2002). A two-stage, p16(INK4A)- and p53-dependent keratinocyte senescence mechanism that limits replicative potential independent of telomere status. Mol Cell Biol. 22, 5157-5172.
